# Microbiome Dynamics and Functional Composition in *Coelopa frigida* (Diptera, Coelopidae): Insights into Trophic Specialization of Kelp Flies

**DOI:** 10.1007/s00248-024-02403-1

**Published:** 2024-07-03

**Authors:** Paul S. P. Bischof, Theda U. P. Bartolomaeus, Ulrike Löber, Christoph Bleidorn

**Affiliations:** 1 Department for Animal Evolution and Biodiversity, Georg-August-Universität Göttingen, Göttingen, Germany; 2 Charité-Universitätsmedizin Berlin, Corporate Member of Freie Universität Berlin, Humboldt-Universität Zu Berlin, and Berlin Institute of Health, Berlin, Germany; 3 Experimental and Clinical Research Center, A Cooperation of Charité-Universitätsmedizin Berlin and Max Delbrück Center for Molecular Medicine, Berlin, Germany; 4 Max Delbrück Center for Molecular Medicine in the Helmholtz Association, Berlin, Germany; 5 German Centre for Cardiovascular Research, Berlin, Germany; 6 Departamento de Biodiversidad y Biología Evolutiva, Museo Nacional de Ciencias Naturales (MNCN-CSIC), Madrid, Spain; 7 Biologische Anstalt Helgoland, Alfred Wegener Institute, Helgoland, Germany

**Keywords:** *Coelopa frigida*, Kelp flies, Metabarcoding

## Abstract

Coelopidae (Diptera), known as kelp flies, exhibit an ecological association with beached kelp and other rotting seaweeds. This unique trophic specialization necessitates significant adaptations to overcome the limitations of an algal diet. We aimed to investigate whether the flies’ microbiome could be one of these adaptive mechanisms. Our analysis focused on assessing composition and diversity of adult and larval microbiota of the kelp fly *Coelopa frigida*. Feeding habits of the larvae of this species have been subject of numerous studies, with debates whether they directly consume kelp or primarily feed on associated bacteria. By using a 16S rRNA metabarcoding approach, we found that the larval microbiota displayed considerably less diversity than adults, heavily dominated by only four operational taxonomic units (OTUs). Phylogenetic placement recovered the most dominant OTU of the larval microbiome, which is the source of more than half of all metabarcoding sequence reads, as an undescribed genus of Orbaceae (Gammaproteobacteria). Interestingly, this OTU is barely found among the 15 most abundant taxa of the adult microbiome, where it is responsible for less than 2% of the metabarcoding sequence reads. The other three OTUs dominating the larval microbiome have been assigned as *Psychrobacter* (Gammaproteobacteria), *Wohlfahrtiimonas* (Gammaproteobacteria), and *Cetobacterium* (Fusobacteriota). Moreover, we also uncovered a distinct shift in the functional composition between the larval and adult stages, where our taxonomic profiling suggests a significant decrease in functional diversity in larval samples. Our study offers insights into the microbiome dynamics and functional composition of *Coelopa frigida*.

**Supplementary Information:**

The online version contains supplementary material available at 10.1007/s00248-024-02403-1.

## Introduction

Novel insights into the broad range of animal–bacterial interactions have fundamentally transformed our understanding of animal biology and evolution [1]. The microbiome expands the genetic repertoire of the host and influences the heritability of its traits, which means it can be regarded as an extended phenotype sensu Dawkins [2]. In the case of insects, bacterial symbionts and other microbes that live inside them play critical roles in host physiology, development, immunity, behavior, and nutrition [3, 4]. Not surprisingly, bacterial symbiosis often enables highly specialized lifestyles of insects [5]. Prominent examples are known from blood-feeding bedbugs [6], plant sap-feeding aphids [7], or wood-feeding termites [8]. Kelp flies (Diptera, Coelopidae), a globally distributed taxon of Diptera containing only 29 species [9], have a rather unique ecological association with unattached seaweeds, primarily residing on wrack beds of marine beaches [10]. They deposit their eggs on beached kelp or other seaweeds, and their larvae feed inside the algae while adults reside and copulate under and on top of the wrack beds [11]. Kelp flies can reach large population sizes and thereby also be utilized as an important food source for a variety of sea birds [12]. The evolutionary significance of these flies lies in their ability to utilize washed-up seaweed as a resource despite its low utilization by other fauna [13, 14]. Kelp and similar seaweeds present challenges due to their physical and chemical properties, such as hard-to-digest polysaccharides, potentially toxic secondary metabolites, and high concentrations of heavy metals [15–19]. However, these wrack beds are known to constitute biogeochemical hotspots due to high metabolic activity by bacteria [20, 21]. Bacterial decomposers thrive within the kelp wrack beds, creating an ideal environment for kelp fly larvae and adults, where the larvae graze on the surrounding kelp [11]. In contrast, adults imbibe fluid from the kelp surface [11].

The best-investigated kelp fly species is *Coelopa frigida*, which occurs on most shorelines in the temperate Northern Hemisphere [9]. Earlier studies on *Coelopa frigida* mainly focused on its mating behavior, genetics, and the inversion polymorphism on chromosome 1, which is responsible for large size differences among adults [22–25]. The chromosomal inversion polymorphism in *Coelopa frigida* results in a threefold size difference in males, while female size is less affected. A reciprocal transplant study demonstrated that the phenotypic size effect in males can be further modulated by environmental variation [26]. Interestingly, the three different inversion karyotypes also seem to have adaptive advantages in different microhabitats, which could explain how this diversity of phenotypes is maintained by balancing selection [27].

As typical for kelp flies, *Coelopa frigida* depends on wrack-beds throughout its life history [28]. It is well adapted to the harsh conditions occurring in this dynamic habitat [28]. The whole life cycle follows the formation and depletion of wrack beds and can be completed in roughly 2 weeks [29]. The females are attracted to freshly deposited seaweed and lay batches of up to 80 eggs into the deeper layers of the wrack bed [30]. The larvae’s feeding habits have also been subject of numerous studies, with debates regarding their preference for specific seaweed species and whether they directly consume kelp or primarily feed on associated bacteria [11, 31–33]. The most thorough investigation of the dietary requirements of *Coelopa frigida* was conducted by Cullen et al., who confirmed the need for microbial colonization of the larval gut and isolated around 20 species of bacteria [11]. Additionally, this study demonstrated that the larvae actively filter bacteria from their environment and can be reared on a medium composed of seaweed and only one bacterial species. This study got additional support from a metabarcoding analysis comparing the microbiome of the larvae with the environmental wrack bed microbiome along selected sampling sites in the North and Baltic Seas [31]. Larval microbiome changes across the investigated gradient correlated with a shift in the microbial community across the sampling sites. Moreover, functional analyses revealed that polysaccharide degraders dominate the larval microbiome [31]. However, Biancarosa et al. showed the limitations of the algal food source for some essential amino acids (e.g., histidine and methionine), which is also reflected by the lower amount of these amino acids in the larvae [34], a deficit often coped with by taking advantage of bacterial symbionts in other insects [35]. The adult diet is not as thoroughly understood, but observations suggest fluid intake from kelp surfaces and nectar intake from visiting flowers [36]. To our knowledge, no studies on the adult microbiome have been published. As typical for all Diptera, a taxon belonging to the holometabolous insects, in Coelopidae, complete metamorphosis occurs during their life cycle, consisting of egg, larva, pupa, and adult stages [36]. In holometabolous insects, due to the renewal of the gut epithelium of their host, the associated microorganisms have to face difficult conditions during metamorphosis, as well as new conditions in the adult insect [37], which is often accompanied by a complete shift of the microbiome community [38]. Simultaneously, this allows symbiotic associations to be decoupled over development [39].

This study focuses on the microbiome shift occurring during the transition from larva to adult in *Coelopa frigida*, hypothesizing that a shift in taxonomic and functional properties of the microbiome accompanies this process. The main objective of this research is to investigate the presence of dietary symbionts in coelopids, using a *Coelopa frigida* population from Helgoland (Germany) as a model. This will serve as a foundation for further investigations into the insect-bacteria relationship and provide insights into the nature of this relationship during the transformation from larva to adult. Studying the microbiome of the kelp fly can provide insights into their unique adaptations for utilizing washed-up kelp as a resource and help understand the complex interactions between insects and their microbial symbionts.

## Methods

### Sample Collection

Samples were collected from the northern beach (Nordstrand) of the North Sea island of Helgoland (Germany) on March 22nd and 23rd, 2022. The beach featured large amounts of unrooted kelp, predominantly from the genus *Laminaria*. Adult kelp flies were primarily collected by hand (using gloves) from the top and bottom of kelp stacks, as their mobility was limited under the low spring temperatures. In contrast, larvae were collected from the deeper, moister layers of the wrack beds. Adults and larvae were transferred to separate collection tubes on site and later stored in a refrigerator at + 5 °C. On-site storage did not exceed 3 h, and refrigerated storage before fixation did not exceed 60 h. No flies died during the storage period. The sex of adult flies was determined by examining genital structures before fixation. Then, 48 larvae, 26 adult males, and 23 adult females were collected.

To best preserve the flies’ microbiome, larvae and adults were submerged in PBS buffer, briefly placed on a laboratory wipe to dry, and then individually placed in Eppendorf tubes containing 1 ml of DNA/RNA Shield™ (Zymo Research). Due to their limited mobility, larvae could be processed alive, while adult flies were immobilized by placing tubes of small batches of flies in the freezer for no longer than 10 min. Flies and larvae were sorted according to their size, with small, medium, and large larvae likely corresponding to the 1st, 2nd, and 3rd instars, respectively. Adults of each sex were also grouped into three sizes. Females showed considerably less variation regarding size, especially towards the higher end of the spectrum. On the contrary, males made up both the largest and smallest, as well as a range of intermediate-sized individuals. This phenomenon led earlier researchers to describe them as three different species [40]. Sizes were sorted relative to each other, meaning large individuals correspond to the upper third of observed sizes among larvae, adult males, and adult females.

### DNA Extraction and 16S rRNA Gene Sequencing

All laboratory procedures were conducted under a laminar flow hood (LabGard ES Energy Saver Class II Laminar Flow, NuAire Inc., Plymouth, MN, USA) to minimize environmental contamination. Total DNA was extracted using the ZymoBIOMICS DNA Miniprep Kit (Zymo Research) following the manufacturer’s instructions. To obtain sufficient microbial DNA, samples were pooled: small flies, 3 per tube; medium larvae, 3 per tube; and small larvae, 4 per tube. Large individuals and medium adults were extracted individually. Adult wings were removed before processing to facilitate tissue breakdown.

The DNA extraction protocol was modified to include an extra bead-beating step using silica beads for efficient insect tissue breakdown. Additionally, blank samples were included during plain water samples’ collection, extraction, and 16S rRNA gene sequencing to identify potential kit contaminants (Fig. S1) [41, 42]. DNA yields were determined using NanoDrop (Thermo Fisher) and stored at – 20 °C before shipping to LGC Genomics Berlin (https://www.lgcgroup.com) for 16S rRNA gene amplification and sequencing. Samples were shipped on dry ice, and vials were pseudonymized before shipping.

The V3–V4 hypervariable region of the 16S rRNA gene was PCR amplified using 16S rRNA-specific primers 341F “Klindworth” (CCTACGGGNGGCWGCAG) and 785R “Klindworth” (GACTACHVGGGTATCTAAKCC). Unique 10-nucleotide barcodes were incorporated into the forward primer for each sample. PCRs were carried out for 30 cycles, and amplicon DNA concentrations were assessed by gel electrophoresis. Approximately 20 ng of amplicon DNA from each sample was pooled and purified using Agencourt AMPure XP beads (Beckman Coulter, Inc., IN, USA) and MinElute columns (Qiagen GmbH, Hilden, Germany).

Illumina libraries were constructed using the Ovation Rapid DR multiplex system 1–96 (NuGEN Technologies, Inc., CA, USA) with approximately 100 ng of purified amplicon pool DNA. Libraries were pooled, size-selected by preparative gel electrophoresis, and sequenced on the Illumina MiSeq platform targeting the V3–V4 region (300-bp read length, paired-end protocol). A total of 50 samples were sequenced, including 18 larval samples, 14 adult males, 13 adult females, and five blind samples.

#### Sample Preparation and Human Contamination Removal

The raw sequences were first processed to eliminate potential host and human contamination following the method described by Kayongo et al. [43]. Briefly, the kelp fly genome (GCA_017309665.1) and human genome (GCF_000001405.39) were masked using the proGenomes2 microbial genome database [44]. Raw reads were mapped to the reference genomes using BBMap (minimum identity of 0.95, maximum indel of 3, bandwidth rate of 0.16, bandwidth of 12, quick match, fast processing, and minimum hits of 2), while read mapping the references was discarded.

#### Sequence Processing and Taxonomic Assignment

After removing putative off-target amplicons, the remaining raw reads were processed using LotuS2 (version 2.16) [45]. A Poisson binomial model–based read filtering was applied [46]. Operational taxonomic unit (OTU) clustering was performed using UPARSE, based on a 97% sequence similarity threshold [47]. The SILVA database v138 used Lambda for taxonomic assignment [48].

### Phylogenetic Analysis

Our phylogenetic analysis focused on two of the most prevalent OTUs (OTU1 unknown genera and OTU3 unknown genera) in the larval samples. The OTUs were not classified beyond the family level by the LotuS2 pipeline, where OTU1 was classified as Orbaceae and OTU3 as Wohlfahrtiimonadaceae. We included the respective sequences in recently published 16S rRNA gene datasets dealing with the phylogeny of Orbaceae and Wohlfahrtiimonadaceae [49, 50]. The alignments were performed with MAFFT version 7 using the FFT-NS-i iterative refinement method [51]. Maximum likelihood analysis was conducted using IQ-TREE 1.6.12 using ModelFinder and 1000 ultrafast bootstrap replicates [52–54].

### Statistical Analysis

Normalization and computation of alpha diversity measures were performed using the rarefaction tool kit (RTK 0.93.1) with default settings [55]. To standardize for differences in sequencing depth in counts of OTUs in the Coelopidae microbiota, we first removed all blank samples. Subsequently, we normalized the remaining samples by rarefying them to the minimum observed read count of 14,515.

Three samples were removed and did not pass the read count threshold (Fig. S5). Due to the non-normal data distribution, only non-parametric statistical tests were utilized for association analyses. Significance levels were established at a *p*-value < 0.05 or a *q*-value (FDR-corrected *p*-value) < 0.1 for multiple testing scenarios. Bray–Curtis dissimilarities for beta diversity were calculated using the vegan R package (version 2.5–7) [56]. Permutational multivariate analysis of variance (PERMANOVA) was employed to evaluate the influence of life stages (adults and larvae), sexes (male and female), and size categories (small, medium, and large) on the Coelopidae microbiome composition using Bray–Curtis distances.

To assess the homogeneity of multivariate dispersions, a prerequisite for PERMANOVA, we applied the betadine function from the vegan package in R and evaluated the spread of multivariate data within groups. Upon detecting significant dispersion differences among life stage groups, we adjusted our PERMANOVA model to include distances to group centroids as a covariate, using the adonis function from the same package.

To evaluate whether the differences in unique taxa across life stages, sexes, and size categories were significant, we performed a permutation test in R. We set a seed for reproducibility, executed 10,000 permutations by shuffling group labels, and generated a null distribution (see Supplementary Figure S3). The significance was determined by calculating a *p*-value from the observed differences compared to this distribution.

The ANCOMBC package in R (version 1.4.0) was utilized to assess the differential abundance of microbial taxa across insect life stages and within-group characteristics. This method accounts for the compositional nature of microbiome data and includes bias correction [57, 58]. We compared microbial abundances using variables such as “type,” “sex,” and “size,” with the Benjamini–Hochberg procedure applied to adjust for multiple testing. Data filtering was based on non-zero count proportions and library sizes. The analysis yielded beta coefficients and *q*-values, indicating the relative abundance changes and their significance, which were compiled for comparative analysis.

Functional profiling of the Coelopidae microbiota, derived from 16S rRNA gene sequence data, was conducted using PICRUSt2 (phylogenetic investigation of communities by reconstructing unobserved states) version 2.2.3 [59]. This method leveraged marker gene data and a reference genome database with 16S rRNA gene sequences to infer functional profiles. The same analytical approach was consistently applied across all Coelopidae samples.

## Results

### Microbiome Composition Variation Across Life Stages, Sexes, and Sizes

**Fig. 1:**
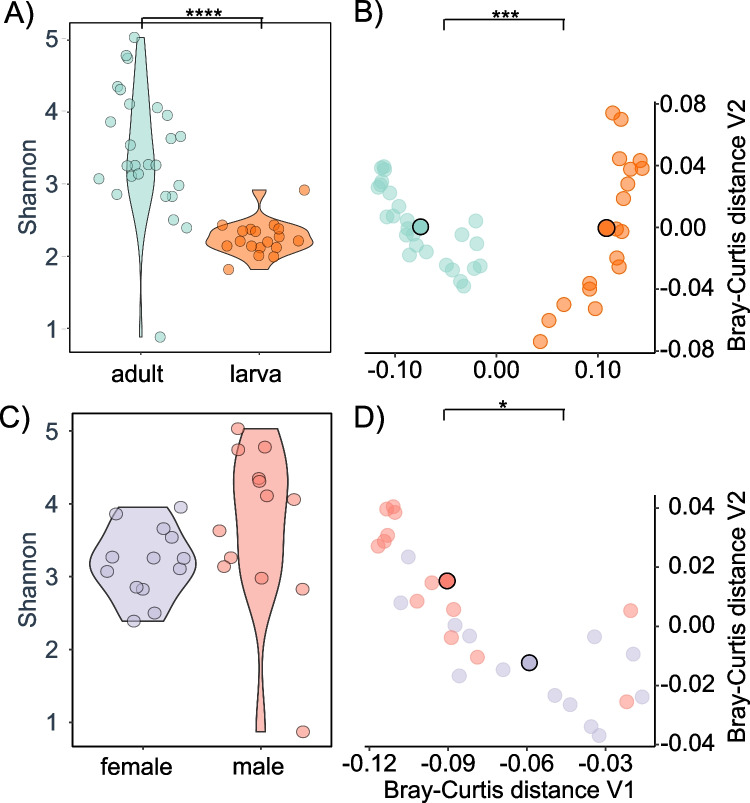
Comparison of diversity indices and dissimilarities between life stages and sexes.** A** Shannon diversity indices show a significant difference between life stages (larva and adult) (Wilcoxon test, *q* < 0.0001, BH-FDR corrected). **B** Intersample Bray–Curtis dissimilarities reveal significant differences between life stages (PERMANOVA, *p* < 0.001). **C** Shannon diversity indices between sexes show no significant difference (Wilcoxon test, BH-FDR corrected). **D** Intersample Bray–Curtis dissimilarities between sexes indicate significant differences (PERMANOVA, *p* < 0.05). Black-bordered points indicate the centroids of the displayed groups

A comparative analysis of Shannon diversity indices was conducted to examine differences in microbiome composition between life stages (adult and larva). The results revealed a highly significant dissimilarity in microbiome composition (Wilcox *p* < 0.0001, BH-FDR) (Fig. 1A). However, contrasting the sexes (male and female) within adults (Fig. 1C) and examining size differences (large, medium, and small) within adults and larvae did not yield statistically significant results (Fig. S2), suggesting that the microbial diversity and composition are consistent across different sizes within each life stage.

To comprehensively assess microbiome composition, intersample Bray–Curtis dissimilarities were determined, reducing the high-dimensional data into a more concise dimensional space. Principal coordinate analysis (PCoA) was employed to visualize the Bray–Curtis dissimilarity between different groups (Fig. 1B: different life stages; Fig. 1C: different sexes within adult animals). Multivariate tests utilizing the Bray–Curtis distance confirmed significant differences in composition across life stages (PERMANOVA *p* < 0.001) and sexes (PERMANOVA *p* < 0.05). Notably, the analysis of variance revealed that 34% of the variance could be attributed to life stage (larva vs. adult), while 4% was explained by sex (Fig. 1C, D). Additionally, the difference in microbial composition was significant concerning size (large, medium, and small) among adult and larval animals (Fig. S2).

**Fig. 2:**
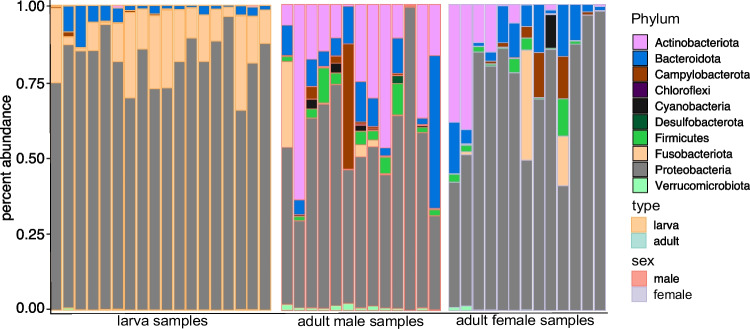
Microbial community composition across different samples. Barplots display the percent abundance of various taxonomic groups across a range of samples, differentiated by life stage (larva and adult) and further categorized by type and sex within the adult samples. The color key indicates the specific phyla, and the shading intensity reflects their relative abundance in each sample

The abundance of bacterial phyla in the dataset between the two life stages, larvae and adults, predominantly comprises Proteobacteria (Fig. 2). The larval samples comprise three dominant phyla: Proteobacteria, Bacteroidota, and Fusobacteria (Table S1). The adult samples, on the other hand, are composed of six dominant phyla, with Proteobacteria and Actinobacteria being particularly abundant, followed by Campylobacter (Table S1).

When analyzing the abundance of phyla for a subset of adult insects categorized by sex and size and a subset of larvae categorized by size, effects of sex and size on the distribution of phyla were observed (Fig. S4, Table S1).

**Fig. 3:**
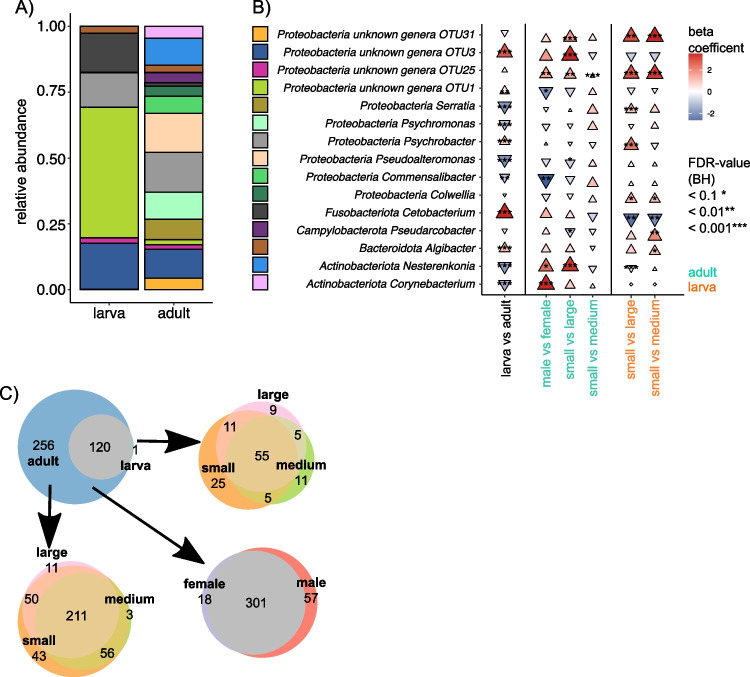
Taxonomic composition analysis and unique taxa across life stages, sexes, and sizes. **A** Barplots illustrating the distribution of the 15 most abundant taxa for two life stages (larva and adult). **B** ANCOMBC analysis of the 15 most abundant taxa, with shape, size, direction, and color indicating effect size (Cliff’s delta) for different comparisons (life stage, sex, and size). Stars represent significance (BH-FDR corrected). **C** Venn diagram illustrates shared and unique taxa between life stages, sizes, and sexes. Permutation testing suggests non-random differences in the number of unique genera between life stages and sizes, except for the sex comparison among adult insects

Visualizing the 15 most abundant taxa in our dataset, the larva samples are predominated by four taxa (OTU1 unknown genus, OTU3 unknown genus, OTU11 *Psychrobacter*, OTU4 C*etobacterium*), with half of the reads stemming from OTU1 unknown genus (Fig. 3A). In contrast, the adult samples exhibit a more even distribution of the top 15 genera (Table S2). Analyzing the same genera for a subset of adult animals categorized by sex and size and a subset of larvae categorized by size also showed a fewer skews in the distribution of the microbiome compared to the effect of life stage (Fig. S5, Table S2).

In our study, we utilized ANCOMBC on non-rarefied data to draw inferences regarding taxon abundance across different life stages (adult and larva), as well as among different sexes and sizes within adult and larval insects. The ANCOMBC approach was chosen due to its robustness and sensitivity, particularly with non-rarefied data and small sample sizes.

The ANCOMBC analysis revealed significant differences in the microbial communities between larval and adult insects (Fig. 3B). Specifically, we identified significant increases in the abundance of Proteobacteria unknown genera OTU1 (*q*-value < 0.01, beta estimate = 1.63), Proteobacteria unknown genera OTU3 (*q*-value < 0.001, beta estimate = 5.74), and Fusobacteriota *Cetobacterium* (*q*-value < 0.001, beta estimate = 6.95) between larva and adult insects. Notably, adult male flies exhibited an increase in Actinobacteriota *Corynebacterium* (*q*-value < 0.001, beta estimate = 3.02).

Our exploration into distinct taxa across life stages, sexes, and sizes revealed intriguing findings. While 120 genera were shared between life stages, adult animals exhibited 256 unique genera, with only one unique genus found in larvae (Fig. 3C).

Permutation testing substantiated a non-random distinction in unique genera between larvae and adults (*p*-value < 0.0001). Comparison of sexes and sizes among adult organisms revealed an overlap of 301 genera, with 18 exclusive to females and 57 to males (Fig. 3C). Evaluation of the distinction between adult males and females yielded a *p*-value < 0.05, suggesting a significant difference in the number of unique genera between sexes, thus indicating that the observed variances are highly improbable to have arisen by chance (Fig. S3).

Lastly, we investigated the abundance of these unique genera. While the single unique genus in the larvae (OTU783 unknown genus) exhibited minimal abundance, the unique genus Bacteroidota *Barnesiella*, Firmicutes *Irregularibacter*, and Proteobacteria *uncultured rhizobia bacterium* constituted together with 15 other a substantial portion of the lowest abundance unique genera in the adult insects (Fig. S6, Table S3).

### Functional Profiling and Diversity Analysis of Microbiota Across Life Stages and Sexes

**Fig. 4:**
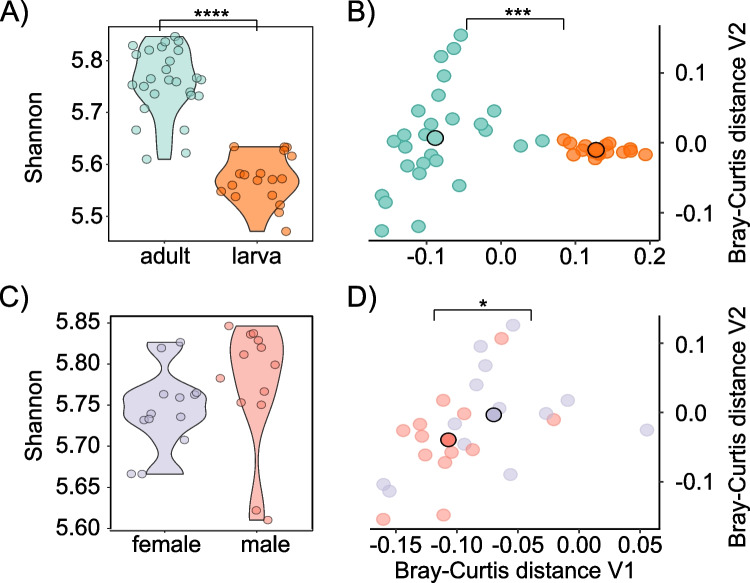
Analysis of functional modules and unique functions in the microbiome using PICRUSt2.** A** Shannon diversity of functional profiles across life stages (larva and adult), with significant differences observed (Wilcoxon test, *q* < 0.001, BH-FDR corrected). **B** Intersample Bray–Curtis dissimilarities reveal significant differences in functional composition across life stages (PERMANOVA, *p* < 0.001). **C** Shannon diversity of the functional profiles across sexes shows no significant difference. **D** Intersample Bray–Curtis dissimilarities between sexes indicate significant differences (PERMANOVA, *p* < 0.05). Black-bordered points indicate the centroids of the displayed groups

To gain insights into the functional profiles of the insect microbiota using 16S rRNA gene data, we employed PICRUSt2. This tool infers the functional composition of bacterial communities based on their taxonomic composition. We assessed the Shannon diversity of the functional profiles across life stages and sexes (Fig. 4A, C). The results demonstrated a significant decrease in functional diversity in the larva samples compared to the adult samples (*q* < 0.001, FDR corrected). However, no significant difference in functional diversity was observed when comparing the sexes within the adult subset (Fig. 4C). Additionally, we observed significant differences in functional composition across life stages (PERMANOVA *p* < 0.001) and sexes (PERMANOVA *p* < 0.05), as indicated by intersample Bray–Curtis dissimilarities (Fig. 4B, D).

### Analysis of Functional Modules and Unique Functions in Microbiota Across Life Stages and Sizes

**Fig. 5:**
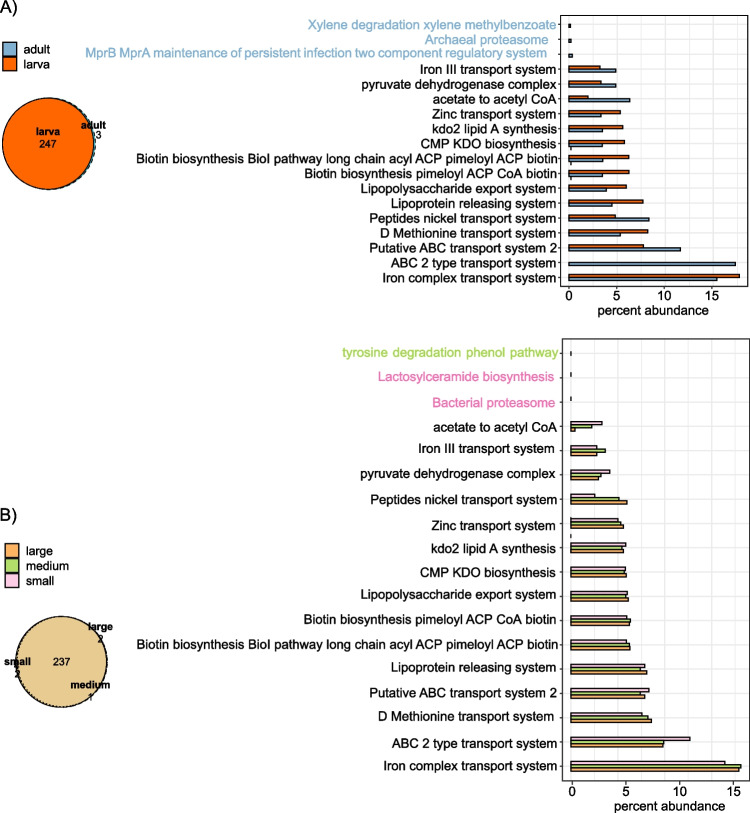
Abundance and unique functions of KEGG and GMM modules across life stages and sizes.** A** Barplots illustrating the differences in the abundance of the 15 most associated KEGG and GMM (Gene Ontology Molecular Function) modules across various life stages (larva and adult). **B** Barplots showing the differences in the abundance of the 15 most associated KEGG and GMM modules across different sizes (small, medium, and large). **A** Venn diagram displays unique functions specific to each life stage among the 250 KEGG and GMM modules, with three modules found to be uniquely present in adult animals. **B** In the larval stage, two unique modules were identified for small larvae, and one unique module was identified for medium-sized larvae

Among the 15 most associated KEGG and GMM (Gene Ontology Molecular Function) modules, we investigated the differences in their abundance across different life stages (Fig. 5A, Table S4). The iron complex transport system and ABC type 2 transporter system were found to be the most abundant modules in both life stages. Additionally, we examined unique functions specific to each life stage among the 250 KEGG and GMM modules. We identified three modules uniquely present in adult animals: xylene degradation, archaeal proteasome, and MrpB-MrpA system. Further analysis explored size and sex within the adult insects, revealing no unique modules. Finally, we investigated differences in module abundance among different sizes of the larva. Likewise, for the adult insect, the top 15 modules were the ABC2 transporter and iron complex transporter. Interestingly, small-sized larvae exhibited a module for lactosylceramide biosynthesis and bacterial proteasome, while middle-sized larvae showed a unique module for tyrosine degradation in the phenol pathway (Fig. 5B, Table S4).

### Phylogenetic Placement of Unassigned OTU1

The maximum likelihood phylogeny generated by IQ-TREE placed the unknown genus OTU1 as a sister taxon of all other Orbaceae included in this analysis (Fig. S7), a clade which is altogether supported by 100% bootstrap support. Ingroup relationships of Orbaceae are less well-supported, with the subsequent branching order of OTU1 unknown genus, *Zophobihabitans entericus*, an endosymbiont described from larvae of the darkling beetle *Zophobas morio*, and a well-supported monophyletic group comprising the genera *Orbus*, *Frischella*, and *Gilliamella*.

OTU3 was placed firmly within the genus *Wohlfahrtiimonas*, which is altogether highly supported as monophyletic group (98%). OTU3 is found with a bootstrap support of 93% as a sister group of *Wohlfahrtiimonas chitiniclastica* (Fig. S8).

## Discussion

The gut microbiome is crucial in insect physiology, behavior, and ecology, where symbiotic relationships with gut bacteria have enhanced the limited metabolic capacity of most insects [60]. In this study, we investigated the gut microbiome of *Coelopa frigida* (Diptera, Coelopidae) in two life stages in a population in Helgoland (Germany): larva and adult. Our analysis revealed substantial differences in microbiome diversity (Shannon index) between different life stages, with a highly significant dissimilarity between larval and adult stages. Contrasting the microbiome composition between sexes and sizes within the adults and larvae did not yield statistically significant differences. However, we could detect a significant difference in the abundance of specific genera grouping for size (large, medium, and small) among the adult and larval insects. We discovered that the size of adult and larval specimens significantly affects the abundance of certain bacterial genera, indicating that the size of the species and its associated metabolic capacity play a role in shaping the structure of the microbiome. This variation between different size classes could be attributed to differences in resource utilization, metabolic rates, or other ecological factors [61]. Moreover, consistent with this result, Mérot et al. found that differently sized males of *Coelopa frigida* differ in their fitness across microhabitats [62]. We also observed an influence of sex on the distribution of certain genera, a result that mirrors human microbiome studies [63]. When comparing the sexes among adult insects, we found an overlap of 301 genera, with 18 unique to females and 57 unique to males. This finding aligns with the idea that sex hormones influence the gut microbial composition in various organisms, but further research is necessary to understand this relationship in insects [63, 64]. Our findings highlight the complexity and diversity of bacterial taxa in insects, which can vary across life stages, sexes, and sizes. Understanding these variations can provide valuable insights into the role of gut microbiota in insect development, reproduction, and overall health.

The influence of the developmental stage (adult vs. larva) on the beta diversity of the microbiome in our study aligns with previously published research, reinforcing the hypothesis that the microbiome in holometabolous insects is affected by their complete metamorphosis, which encompasses significant alterations in their physiology, morphology, and ecology [39, 65]. A comparative study found a much higher microbiota turnover in holometabolous insects than in hemimetabolous insects [38]. In the case of *Coelopa frigida* from Helgoland, we find the larval microbiome dominated by Proteobacteria, while the adult microbiome showed a higher variety of phylum-level bacterial taxa. Some OTUs that are predominant in the larval microbiome nearly disappear from the list of the most abundant taxa in adults. The fact can also explain such a strong change in the microbiome composition of many holometabolous insects, which are decoupled, as demonstrated for honey bees [66]. During metamorphosis, most of the larval gut is replaced, which poses a problem for transmitting symbionts across life stages [67]. For example, for several mosquito species, a near-complete elimination of gut bacteria has been found when investigating freshly emerging adults [68]. This problem can be circumvented with the presence of specific gut paunches which facilitate symbiont transmission [69]. Specific investigations targeting metamorphosis are needed to address this question in kelp flies.

Diet, habitat, and host physiology impact insect microbial composition and diversity [70]. Disentangling the effects of dietary change from developmental changes is challenging. Both larval and adult stages of Coelopidae inhabit the kelp, providing them with a food source. These macroalgae contain complex polysaccharides, which can make up to half of their biomass, and specific enzymes are needed for their degradation [71, 72]. A similar study by Berdan and colleagues characterized the microbiome of *Coelopa frigida* larvae and the wrack bed they inhabited across different sampling sites in the North and Baltic Seas [31]. They found that polysaccharide degraders dominated both microbiomes [31]. Differences between wrack bed and fly microbiomes led them to hypothesize that microbes were selected for their abilities to degrade different polysaccharides [31]. Similar to most of the populations investigated by Berdan and colleagues, we found that the major component of the larval microbiome of the Helgoland population is Proteobacteria [31]. We could show that the larval microbiome is dominated by only four OTUs, with around 50% of the metabarcoding reads coming from a single hitherto uncharacterized OTU belonging to the Gammaproteobacteria. We applied phylogenetic analysis to help clarify its taxonomic assignment and placed it as a sister taxon to other included Orbaceae. This family includes mostly taxa found as symbionts of insects like bees or butterflies [69, 73–75]. Additionally, unclassified Orbaceae species were found in fruit flies and beetles [76–78]. The best-studied Orbaceae genus is *Gilliamella*, found in several Hymenoptera with a full-genome analysis indicating they are involved in the degradation of multiple carbohydrates [79]. According to our phylogenetic analysis, the highly abundant OTU1 belongs to an undescribed genus of Orbales, and investigating the complete genome or functional metagenomics would be needed to see if gene encoding for polysaccharide-degrading enzymes are present. While this OTU dominates the larval microbiome, only 2% of the reads of the adult microbiome are assigned to this genus. A not further characterized OTU belonging to Orbaceae also was found in the very high read count numbers in the study by Berdan et al. in the larval samples, but less frequently in the wrack bed samples [31]. Interestingly, with the Actinobacteriota *Demequina* sp., they also report very high read counts for another OTU, which also shows the potential in being beneficial in degrading complex carbon sources [80]. In contrast, we find only very low read counts for Actinobacteriota in our larval samples (Table S1).

Another highly abundant Proteobacteria OTU found in the larval microbiome of *Coelopa frigida* is OTU3, which, based on a detailed phylogenetic analysis, belongs to the genus *Wohlfahrtiimonas* and is closely related to *Wohlfahrtiimonas chitiniclastica*. Around 18% of the metabarcoding reads can be assigned to this OTU for larvae, and with around 11% of metabarcoding reads, this OTU is among the most abundant genera in the more diverse adult microbiome. Berdan and colleagues’ metabarcoding study also revealed this taxon’s presence in the North Sea and Baltic Sea populations of *Coelopa frigida* [31]. Interestingly, in their study, these bacteria were only found in the larval samples, but not in the environmental samples from the wrack bed [31]. The genus *Wohlfahrtiimonas* belongs to the Cardiobacteriales (Gammaproteobacteria) and comprises three described species. The type species *Wohlfahrtiimonas chitiniclastica* was originally isolated from larvae of the spotted flesh-fly *Wohlfahrtia magnifica* (Diptera: Sarcophagidae) and is known to be a zoonotic human pathogen causing, among other problems, sepsis in cases of myasis [81, 82]. *Wohlfahrtiimonas* spp. have been found in the microbiome of several species of Diptera belonging to the Sarcophagidae, Stratiomyidae, Muscidae, or Calliphorida, but also have been isolated from other sources, such as meat, soil, or the bark tissue of a tree canker [49, 82–85]. While several reports about the pathogenesis in humans of *Wohlfahrtiimonas* have been published and the complete genomes are characterized, there seems to be no data on the influence of these bacteria on their insect host [86].

Thirteen percent of the metabarcoding reads of the larval microbiome can be assigned to the genus *Psychrobacter* (Gammaproteobacteria), with a similar number seen in the adult microbiome (15%). Most *Psychrobacter* species are reported from cold environments, but strains are also known from temperate marine environments [87]. *Psychrobacter* is frequently found in studies dealing with the microbiome associated with macroalgae and is also reported from *Laminaria* species [88–90]. Given the distribution of the bacteria, it seems likely that *Coelopa frigida* acquires them from the environment while feeding on the rotten kelp.

Finally, around 15% of the reads of the larval microbiome are mapped to an OTU classified as *Cetobacterium* (Fusobacteriota), which only makes up around 1% of the reads of the adult microbiome. A comparative study on the microbiome of different larval stages of a *Chironomus* sp. (Diptera) found *Cetobacterium* among the most abundant bacterial genera [91]. Functional studies revealed that *Cetobacterium* spp. synthesize vitamin B_12_, which has been shown to improve host resistance against pathogen infection [92, 93]. The exact role of these Fusobacteriota in the microbiome of *Coelopa frigida* remains elusive, but its abundance in larval samples suggests a possible important role. However, while this taxon was also found in the microbiome of some larval samples of the study of Berdan et al. [31], it was recovered much less common and made up less than 1% of the read count.

Adult samples displayed a more even distribution in their microbiome composition, with taxa like *Cetobacterium* disappearing from the list of the 15 most abundant taxa and new taxa like *Pseudoalteromonas*, *Psychromonas*, and *Nesterenkonia* appearing in significantly higher abundances.

To better understand the functional profiles of the microbiota, we analyzed the functional composition of bacterial communities based on their taxonomic composition. Our taxonomic profiling suggests a significant decrease in functional diversity in larva samples compared to adult samples. Similar patterns are also known from other insects, e.g., the fall armyworm *Spodoptera frugiperda* (Lepidoptera) [94]. This reduction can likely be attributed to the overall decreased bacterial richness in larvae, suggesting that the metabolic capacity in larvae may not be as diverse as in adult insects. No significant difference in functional diversity was observed when comparing sexes within the adult subset. We investigated the abundance of the 15 most associated KEGG and GMM (Gene Ontology Molecular Function) modules across different life stages. Overall, the hallmark functions are preserved in the microbiome. We identified three modules uniquely present in adult animals: xylene degradation, archaeal proteasome, and MrpB-MrpA system. However, these did not correspond to abundant OTUs, and it is unlikely they play a vital role. Importantly, the functional predictions made in this study should be interpreted with caution. Most inferences about microbial genes and their functions in PICRUSt2 are based on previous gene annotations. Therefore, any error or limitation in these annotations could impact the accuracy of our functional predictions. Additionally, it is crucial to acknowledge that amplicon-based predictions, like those used in our study, lack the resolution to distinguish strain-specific functionality. This is a significant limitation of PICRUSt2 and any amplicon-based analysis, as they can only differentiate taxa to the extent that they vary at the amplified marker gene sequence. This limitation is critical in understanding the functional dynamics of the microbiome, as it may lead to an underestimation of the true functional diversity present within these communities.

Even though experimental data and microbiome studies are available now for *Coelopa frigida*, several open questions remain regarding their feeding biology: Are they primary consumers, degrading the algal material with the help of bacterial symbionts, or are they secondary consumers feeding on the bacterial component of the algae? The most thorough investigation of the dietary requirements *of* *Coelopa frigida* was done by Cullen and colleagues [11]. They showed that larvae can survive when eggs are externally sterilized, but not if seaweed is sterilized [11]. Cullen and colleagues confirmed the need for microbial colonization of the larval gut and isolated around 20 species of bacteria [11]. The predominant genera are *Bacillus*, *Flavobacterium*, *Staphylococcus*, and possibly *Enterobacter* and *Sarcina*. Additionally, this study showed that the larvae actively filter bacteria from their environment [11]. They can be reared on a medium composed of seaweed and only one bacterial species, even one normally not associated with algae [11]. However, the presented data by Berdan et al. showed that the importance of culturable species was overestimated in their culture-dependent study [31]. A bacterial dietary component of *Coelopa frigida* was also confirmed by comparing the chemical composition and the stable isotopes of flies and algae, and the limitations of the algae food source in the aspect of essential amino acids were likewise demonstrated [34]. In the light of the here presented data and when interpreting the results by Berdan et al., we agree with Cullen et al. that the combined activities of microbial and insect populations result in the rapid decomposition of seaweed, thereby blurring the distinction between primary and secondary consumption in the case of *Coelopa frigida* [11, 31].

## Conclusion

Our study offers insights into the microbiome dynamics and functional composition of *Coelopa frigida*. The results revealed a highly significant dissimilarity in microbiome composition between life stages, with a significant difference in microbial richness and functional diversity between larva and adult samples. However, it is important to note the limitations of our study, as it was conducted in only one location (Helgoland, Germany) with one population of a single kelp fly species during one time of the year. A longitudinal study design would provide the opportunity to investigate the seasonal effects, such as temperature, on the microbiome dynamics and functional composition in *Coelopa frigida*. Future research should consider incorporating a broader range of sampling locations and times to understand better the complex interactions between the kelp fly microbiome, their unique adaptations, and the environmental factors that influence their microbial communities.

### Supplementary Information

Below is the link to the electronic supplementary material.

Supplementary file1 (PDF 1982 KB)

## Data Availability

The data generated for the current study are available in the National Center for Biotechnology Information Sequence Read Archive repository (PRJNA1051845).
