# Supplementary material for: Microbiome Dynamics and Functional Composition in Coelopa frigida (Diptera, Coelopidae): Insights into Trophic Specialization of Kelp Flies
Source: Microb Ecol. 2024 Jul 3;87:91. doi: 10.1007/s00248-024-02403-1 (PMC11222186; doi:10.1007/s00248-024-02403-1)
Supplement: Supplementary file 1 [file 248_2024_2403_MOESM1_ESM.pdf]

Microbiome Dynamics and Functional Composition in *Coelopa frigida* (Diptera, Coelopidae): Insights into Trophic Specialization of Kelp Flies

Paul S. P. Bischof, Theda U.P. Bartolomaeus, Ulrike Löber, and Christoph Bleidorn
Corresponding author: Christoph Bleidorn (christoph.bleidorn@biologie.uni-goettingen.de), Department for Animal Evolution and Biodiversity, Georg-August-Universität Göttingen, Göttingen, Germany

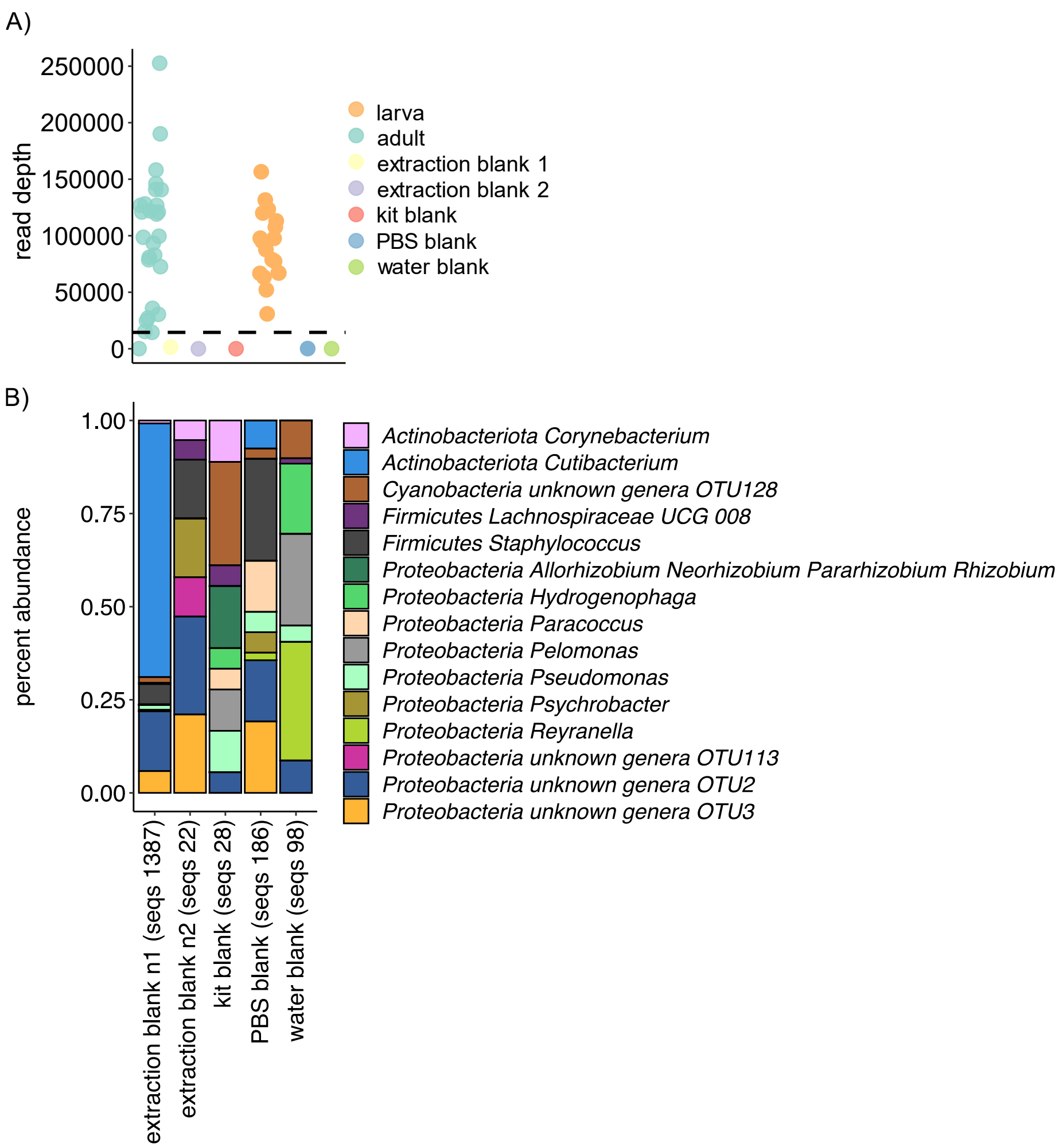

Supplementary Figure 1: Read depth and abundance of the top 15 taxa in control samples. (A) Barplot illustrates the read depth of control samples (collection, extraction, and sequencing controls) compared to insect samples, with samples coloured by origin. (B) Barplot showing the top 15 most abundant taxa in the different control samples, providing insights into potential contamination or background noise in the dataset.

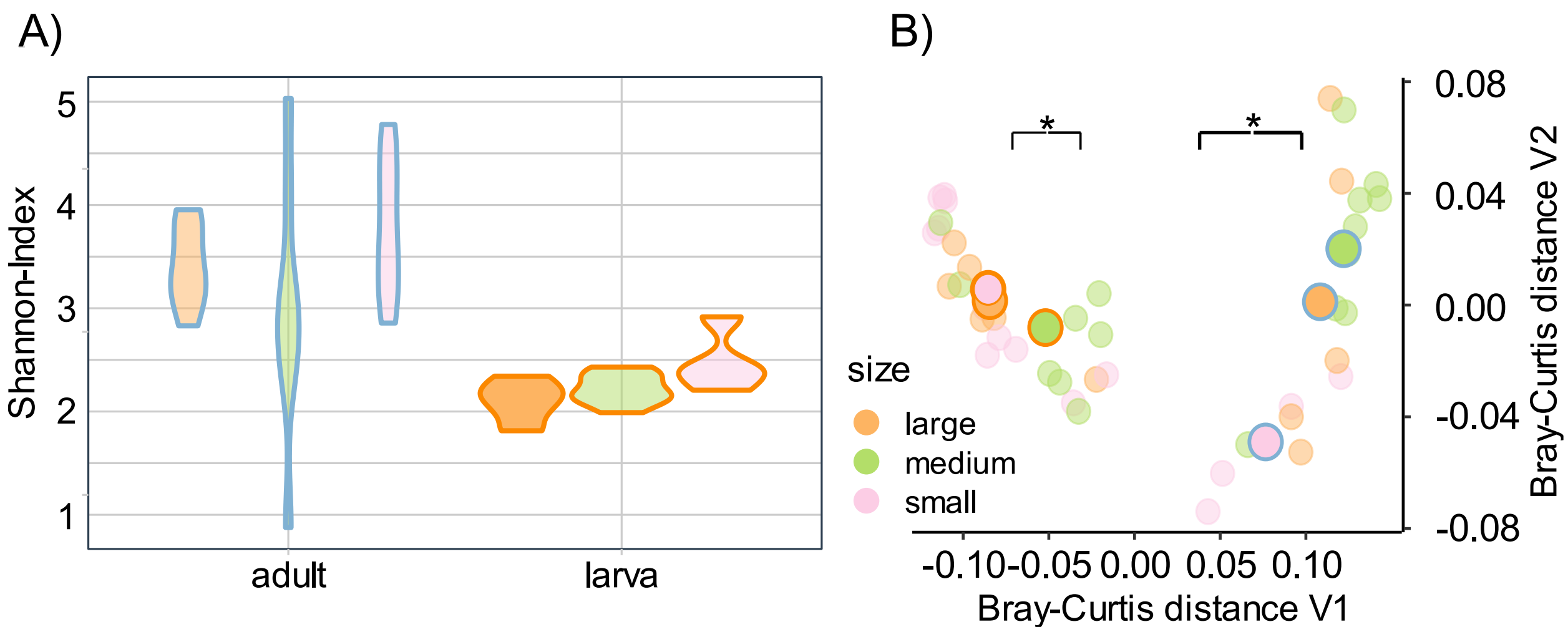

Supplementary Figure 2: Comparison of diversity indices and dissimilarities between sizes in different life stages (larva and adult). (A) Shannon diversity indices show no significant difference between the sizes within the life stages, respectively (BH-FDR corrected). (B) Intersample Bray-Curtis dissimilarities reveal no significant differences between sizes for the larva and adult life stages, respectively. Black-bordered points indicate the centroids of the displayed groups.

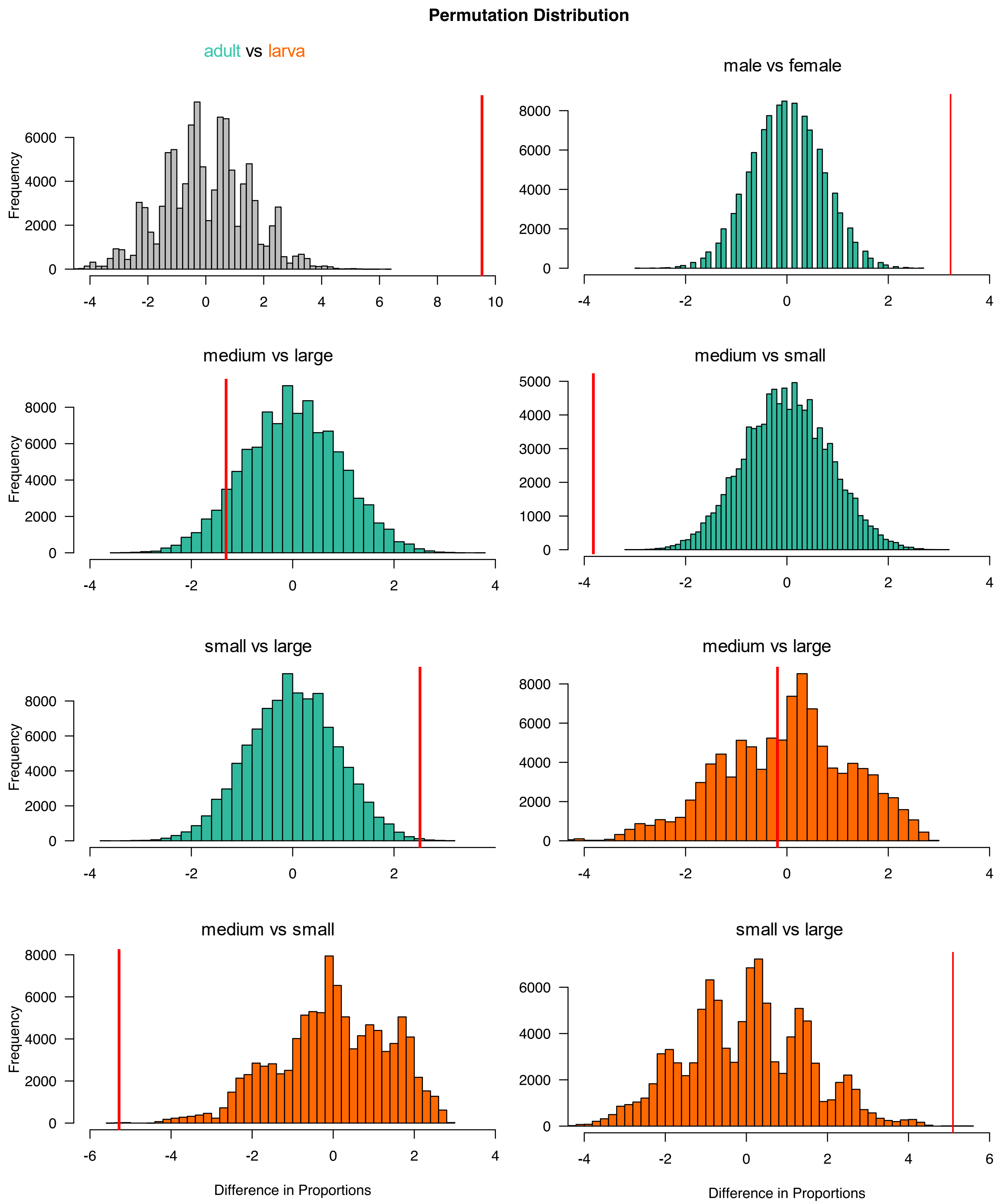

Figure S3: Top 15 most abundant taxa in adult and larval subsets categorized by sex and size. Barplots illustrate the top 15 most abundant taxa for a subset of adult insects categorized by sex (male and female) and size (small, medium, large), as well as a subset of larvae categorized by size (small, medium, large).

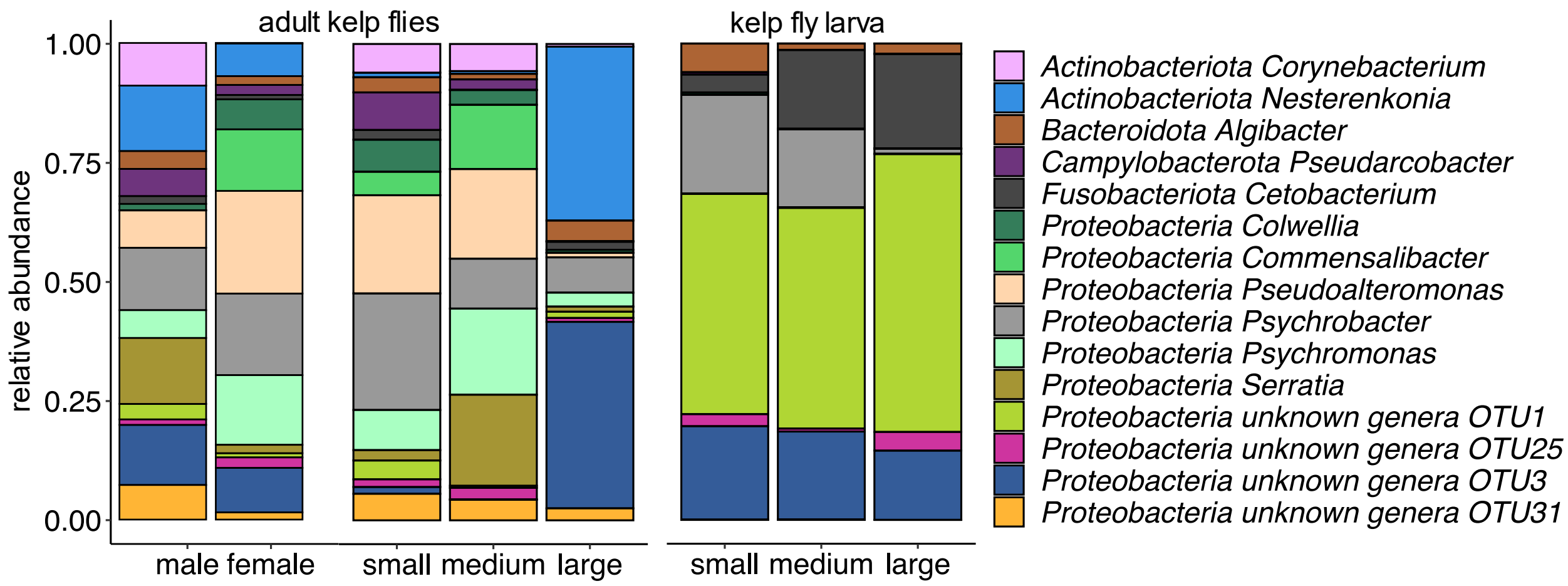

Supplementary Figure 4: Phylum percent abundance abundant in adult and larval subsets categorized by sex and size. Barplots illustrate the Phylum abundance taxa for a subset of adult insects categorized by sex (male and female) and size (small, medium, large), as well as a subset of larvae categorized by size (small, medium, large).

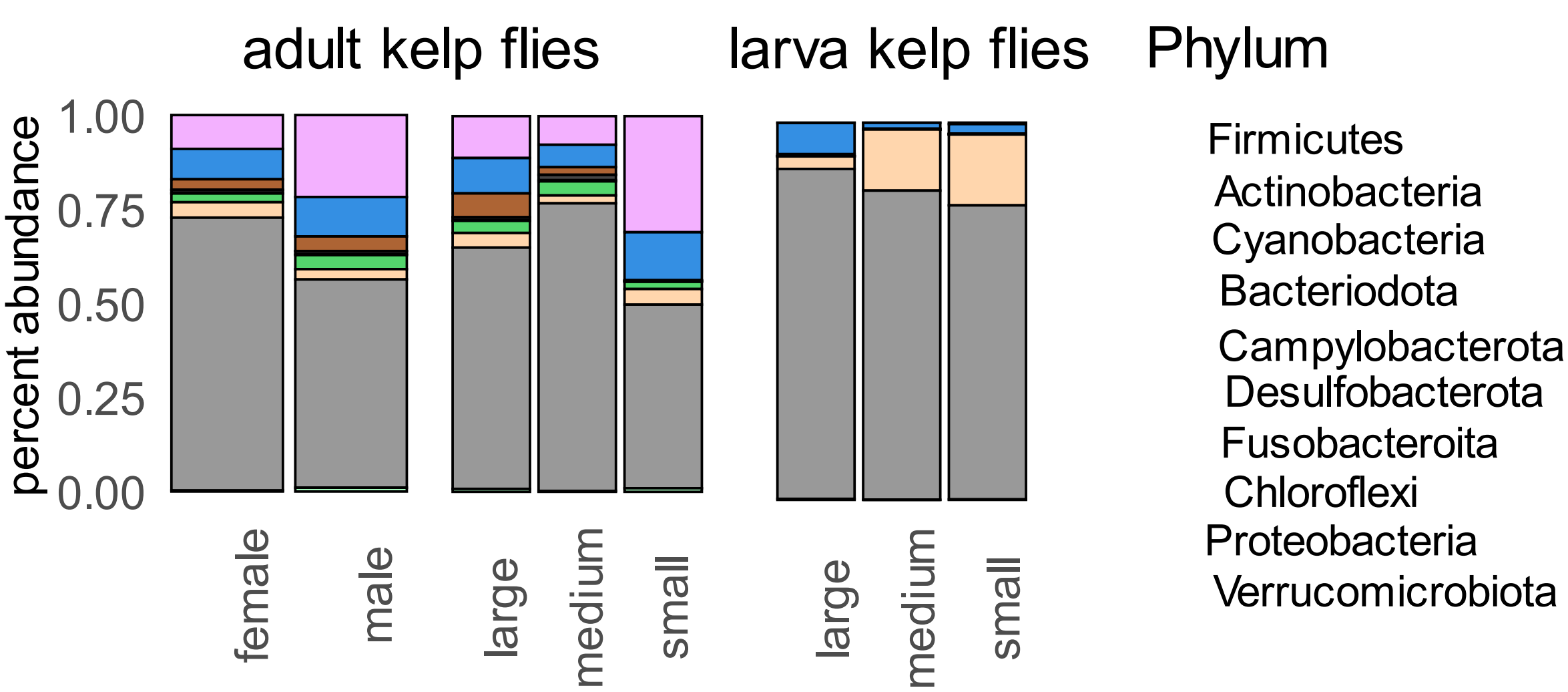

Supplementary Figure 5: Top 15 most abundant taxa in adult and larval subsets categorized by sex and size. Barplots illustrate the top 15 most abundant taxa for a subset of adult insects categorized by sex (male and female) and size (small, medium, large), as well as a subset of larvae categorized by size (small, medium, large).

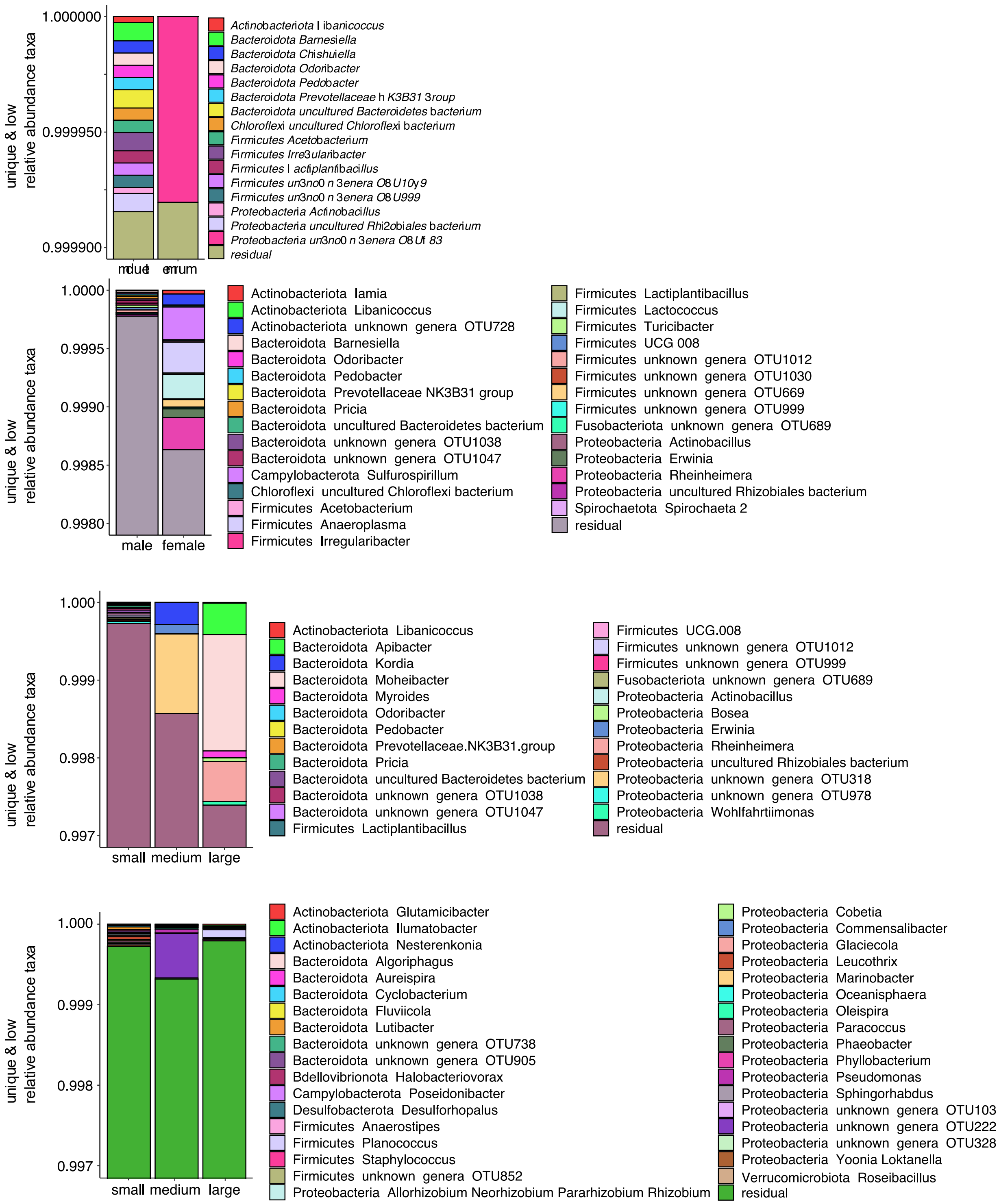

Supplementary Figure 6: Abundance of top 15 unique taxa in adult and larval subsets categorized by sex and size. From top to bottom, boxplots display the abundance of the top 15 unique taxa for life stages (adult and larva) and subset adult insects, which are categorized by sex (male and female) and size (small, medium, large). Boxplots illustrating the abundance of the top 10 unique taxa for larval samples, categorized by size (small, medium, large).

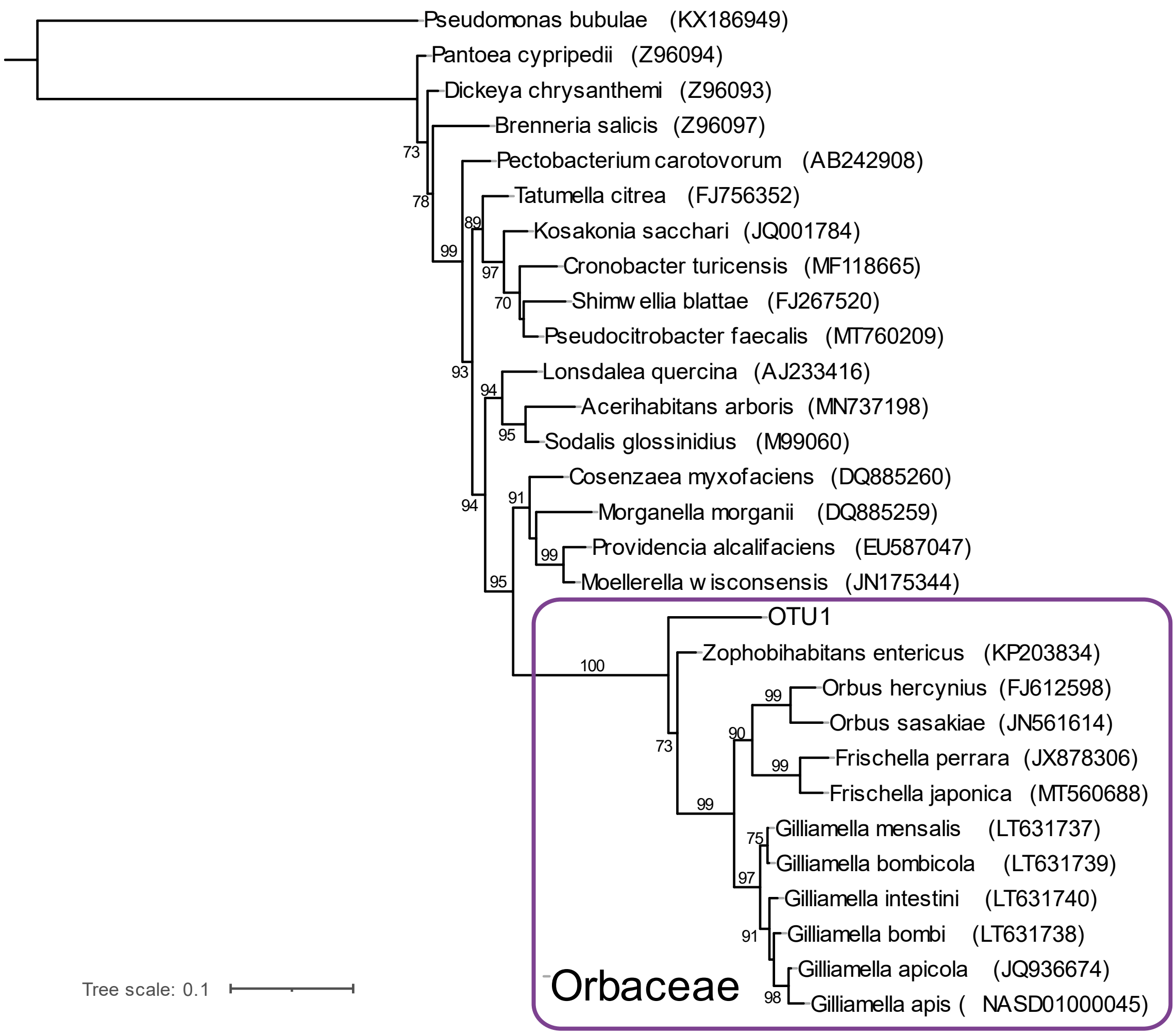

Supplementary Figure 7: Phylogenetic placement of OTU1 unknown genera based on 16S sequence data. Phylogram based on Maximum Likelihood analysis using the TIM3+F+I+G model of sequence evolution. Bootstrap values from 1000 replicates are given at the nodes.

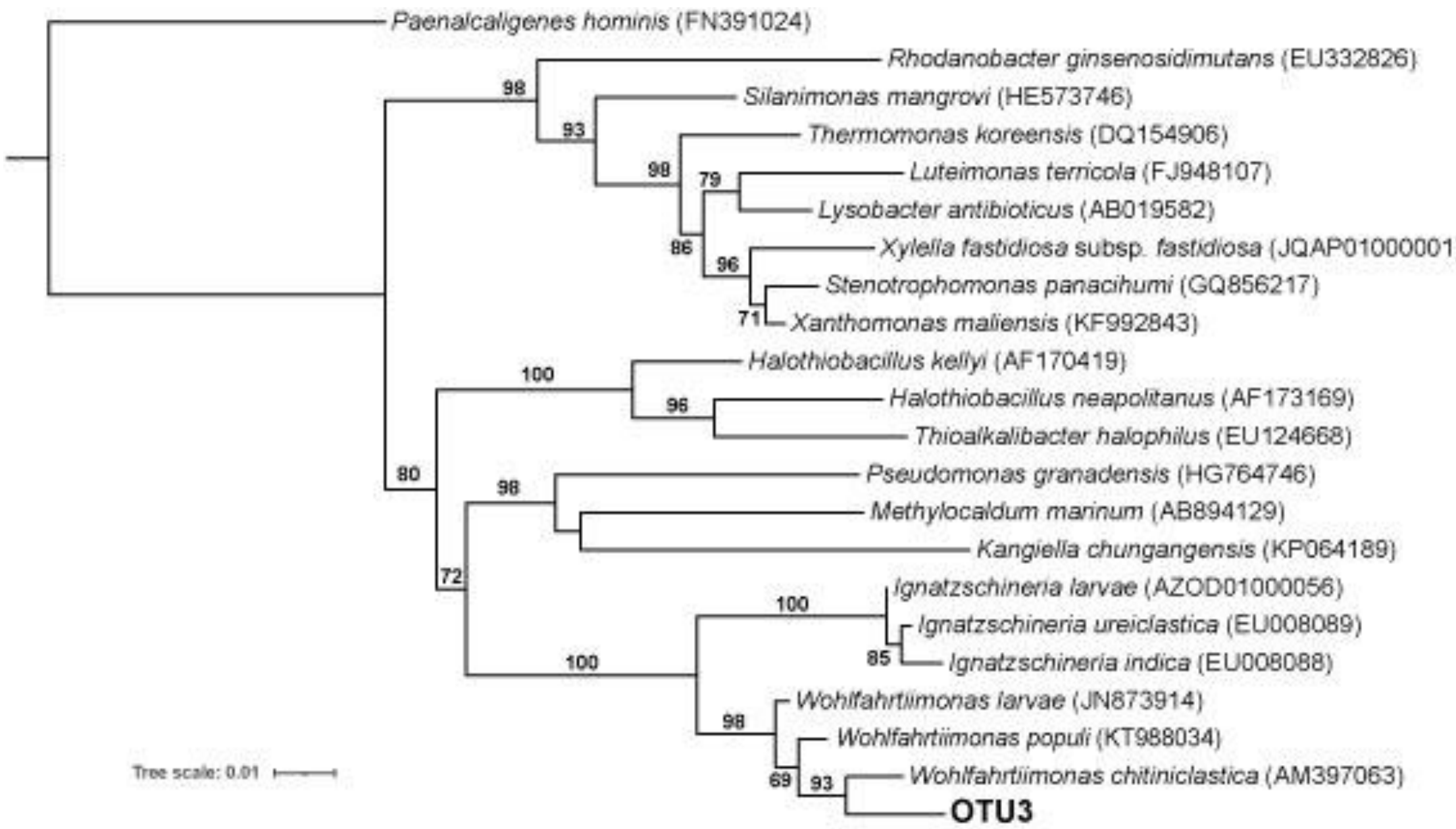

Supplementary Figure 8: Phylogenetic placement of OTU3 unknown genera based on 16S sequence data. Phylogram based on Maximum Likelihood analysis using the TIM3+F+I+G model of sequence evolution. Bootstrap values from 1000 replicates are given at the nodes.

Table S1: Phylum relative and percent abundance

Phylum	life-stage	relative abundance	percent abundance
Proteobacteria	larva	0.82063491	82.06349
Actinobacteriota	larva	0.00151187	0.15119
Cyanobacteria	larva	2.30E-05	0.0023
Bacteroidota	larva	0.03308123	3.30812
Campylobacterota	larva	0.00264099	0.2641
Desulfobacterota	larva	1.53E-05	0.00153
Fusobacteriota	larva	0.14051962	14.05196
Chloroflexi	larva	0	0
Firmicutes	larva	0.00043634	0.04363
Verrucomicrobiota	larva	0.00113677	0.11368
Proteobacteria	adult	0.6389509	63.89509
Actinobacteriota	adult	0.15379333	15.37933
Cyanobacteria	adult	0.0084579	0.84579
Bacteroidota	adult	0.09258091	9.25809
Campylobacterota	adult	0.03367784	3.36778
Desulfobacterota	adult	0.00128327	0.12833

Fusobacteriota	adult	0.03417895	3.41789
Chloroflexi	adult	0.00021476	0.02148
Firmicutes	adult	0.03034505	3.03451
Verrucomicrobiota	adult	0.00651709	0.65171
**Sex subset for adult insects only**			
Proteobacteria	female	0.724684467	72.468
Actinobacteriota	female	0.089934216	8.993
Cyanobacteria	female	0.008894826	0.889
Bacteroidota	female	0.080562314	8.056
Campylobacterota	female	0.028428457	2.843
Desulfobacterota	female	0.000238538	0.024
Fusobacteriota	female	0.041012674	4.101
Chloroflexi	female	9.01E-05	0.009
Firmicutes	female	0.023307836	2.331
Verrucomicrobiota	female	0.002846556	0.285
Proteobacteria	male	0.553155485	55.316
Actinobacteriota	male	0.217698514	21.77
Cyanobacteria	male	0.008020667	0.802
Bacteroidota	male	0.10460817	10.461
Campylobacterota	male	0.038931002	3.893
Desulfobacterota	male	0.002328752	0.233
Fusobacteriota	male	0.02734029	2.734
Chloroflexi	male	0.000339499	0.034
Firmicutes	male	0.037387342	3.739
Verrucomicrobiota	male	0.010190279	1.019
**Size subset for adult insects only**			
Proteobacteria	large	0.48870907	48.87091
Actinobacteriota	large	0.30898943	30.89894
Cyanobacteria	large	0.00231331	0.23133
Bacteroidota	large	0.12776367	12.77637
Campylobacterota	large	0.00158486	0.15849
Desulfobacterota	large	0.00041344	0.04134
Fusobacteriota	large	0.04167897	4.1679
Chloroflexi	large	0	0
Firmicutes	large	0.01899868	1.89987
Verrucomicrobiota	large	0.00954856	0.95486
Proteobacteria	medium	0.76577655	76.57766
Actinobacteriota	medium	0.0761995	7.61995
Cyanobacteria	medium	0.0140839	1.40839

Bacteroidota	medium	0.05960031	5.96003
Campylobacterota	medium	0.0204152	2.04152
Desulfobacterota	medium	0.00316995	0.317
Fusobacteriota	medium	0.02097511	2.09751
Chloroflexi	medium	0.0001206	0.01206
Firmicutes	medium	0.03749677	3.74968
Verrucomicrobiota	medium	0.00216212	0.21621
Proteobacteria	small	0.64233224	64.23322
Actinobacteriota	small	0.11142044	11.14204
Cyanobacteria	small	0.00827659	0.82766
Bacteroidota	small	0.09417755	9.41775
Campylobacterota	small	0.06377362	6.37736
Desulfobacterota	small	0.00046399	0.0464
Fusobacteriota	small	0.03901283	3.90128
Chloroflexi	small	0.0004201	0.04201
Firmicutes	small	0.03236648	3.23665
Verrucomicrobiota	small	0.00775617	0.77562

**Size subset for larva only**			
Proteobacteria	large	0.77934246	77.9342
Actinobacteriota	large	0.00378924	0.3789
Cyanobacteria	large	1.38E-05	0.0014
Bacteroidota	large	0.02521564	2.5216
Campylobacterota	large	0.00198418	0.1984
Desulfobacterota	large	1.38E-05	0.0014
Fusobacteriota	large	0.18768429	18.7684
Chloroflexi	large	0	0
Firmicutes	large	0.0005236	0.0524
Verrucomicrobiota	large	0.00143302	0.1433
Proteobacteria	medium	0.81962721	81.9627
Actinobacteriota	medium	0.0004746	0.0475
Cyanobacteria	medium	0	0
Bacteroidota	medium	0.0154859	1.5486
Campylobacterota	medium	0.00180656	0.1807
Desulfobacterota	medium	0	0
Fusobacteriota	medium	0.16205458	16.2055
Chloroflexi	medium	0	0
Firmicutes	medium	0.0003062	0.0306

Verrucomicrobiota	medium	0.00024496	0.0245
Proteobacteria	small	0.87451987	87.452
Actinobacteriota	small	0.00099902	0.0999
Cyanobacteria	small	8.61E-05	0.0086
Bacteroidota	small	0.08250512	8.2505
Campylobacterota	small	0.00533958	0.534
Desulfobacterota	small	5.17E-05	0.0052
Fusobacteriota	small	0.0331054	3.3105
Chloroflexi	small	0	0
Firmicutes	small	0.00062008	0.062
Verrucomicrobiota	small	0.00277314	0.2773

Table S2: Genus relative and percent abundance

**OTU_Genus**	**life-stage**	**relative abundance**	**percent abundance**
Proteobacteria unkown genera OTU1	larva	0.49661332	49.66133
Proteobacteria unkown genera OTU3	larva	0.17618393	17.61839
Proteobacteria Psychrobacter	larva	0.13017028	13.01703
Fusobacteriota Cetobacterium	larva	0.14804565	14.80457
Proteobacteria Pseudoalteromonas	larva	0.00010892	0.01089
Proteobacteria Psychromonas	larva	0.00083104	0.0831
Actinobacteriota Nesterenkonia	larva	4.034e-06	4e-04
Proteobacteria Serratia	larva	4.840e-05	0.00484
Proteobacteria Commensalibacter	larva	4.034e-06	4e-04
Bacteroidota Algibacter	larva	0.02509248	2.50925
Actinobacteriota Corynebacterium	larva	0	0
Proteobacteria unkown genera OTU31	larva	0.00060916	0.06092
Proteobacteria Colwellia	larva	0.00183554	0.18355
Campylobacterota Pseudarcobacter	larva	0.00129093	0.12909
Proteobacteria unkown genera OTU25	larva	0.01916227	1.91623
Proteobacteria unkown genera OTU1	adult	0.02053842	2.05384
Proteobacteria unkown genera OTU3	adult	0.10961055	10.96105
Proteobacteria Psychrobacter	adult	0.15144475	15.14448
Fusobacteriota Cetobacterium	adult	0.01277322	1.27732
Proteobacteria Pseudoalteromonas	adult	0.14781532	14.78153
Proteobacteria Psychromonas	adult	0.10291759	10.29176

Actinobacteriota Nesterenkonia	adult	0.10245806	10.24581
Proteobacteria Serratia	adult	0.07703501	7.7035
Proteobacteria Commensalibacter	adult	0.06589568	6.58957
Bacteroidota Algibacter	adult	0.02788663	2.78866
Actinobacteriota Corynebacterium	adult	0.0446084	4.46084
Proteobacteria unkown genera OTU31	adult	0.04331065	4.33107
Proteobacteria Colwellia	adult	0.03837497	3.8375
Campylobacterota Pseudarcobacter	adult	0.0386175	3.86175
Proteobacteria unkown genera OTU25	adult	0.01671326	1.67133
**Sex subset for adult insects only**			
Proteobacteria unkown genera OTU1	female	0.00887671	0.888
Proteobacteria unkown genera OTU3	female	0.09362872	9.363
Proteobacteria Psychrobacter	female	0.17127266	17.127
Fusobacteriota Cetobacterium	female	0.00926229	0.926
Proteobacteria Pseudoalteromonas	female	0.215279	21.528
Proteobacteria Psychromonas	female	0.14618486	14.618
Actinobacteriota Nesterenkonia	female	0.06864989	6.865
Proteobacteria Serratia	female	0.01756062	1.756
Proteobacteria Commensalibacter	female	0.12928643	12.929
Bacteroidota Algibacter	female	0.01854134	1.854
Actinobacteriota Corynebacterium	female	0.00103101	0.103
Proteobacteria unkown genera OTU31	female	0.01493701	1.494
Proteobacteria Colwellia	female	0.06279914	6.28
Campylobacterota Pseudarcobacter	female	0.02087158	2.087
Proteobacteria unkown genera OTU25	female	0.02181876	2.182
Proteobacteria unkown genera OTU1	male	0.03256079	3.256
Proteobacteria unkown genera OTU3	male	0.12608666	12.609
Proteobacteria Psychrobacter	male	0.13100361	13.1
Fusobacteriota Cetobacterium	male	0.01639273	1.639
Proteobacteria Pseudoalteromonas	male	0.07826515	7.827
Proteobacteria Psychromonas	male	0.05831216	5.831
Actinobacteriota Nesterenkonia	male	0.13731183	13.731
Proteobacteria Serratia	male	0.1383488	13.835
Proteobacteria Commensalibacter	male	0.00054441	0.054
Bacteroidota Algibacter	male	0.03752096	3.752
Actinobacteriota Corynebacterium	male	0.08953354	8.953

Proteobacteria unkown genera OTU31	male	0.07256183	7.256
Proteobacteria Colwellia	male	0.01319542	1.32
Campylobacterota Pseudarcobacter	male	0.05691226	5.691
Proteobacteria unkown genera OTU25	male	0.01144985	1.145
**Size subset for adult insects only**			
Proteobacteria unkown genera OTU1	small	0.03969748	3.9697
Proteobacteria unkown genera OTU3	small	0.01432069	1.4321
Proteobacteria Psychrobacter	small	0.24476849	24.4768
Fusobacteriota Cetobacterium	small	0.02053431	2.0534
Proteobacteria Pseudoalteromonas	small	0.20608303	20.6083
Proteobacteria Psychromonas	small	0.08427009	8.427
Actinobacteriota Nesterenkonia	small	0.0096632	0.9663
Proteobacteria Serratia	small	0.02163339	2.1633
Proteobacteria Commensalibacter	small	0.04940421	4.9404
Bacteroidota Algibacter	small	0.03182981	3.183
Actinobacteriota Corynebacterium	small	0.06023179	6.0232
Proteobacteria unkown genera OTU31	small	0.05574841	5.5748
Proteobacteria Colwellia	small	0.06713096	6.7131
Campylobacterota Pseudarcobacter	small	0.07872028	7.872
Proteobacteria unkown genera OTU25	small	0.01596387	1.5964
Proteobacteria unkown genera OTU1	medium	0.00436454	0.4365
Proteobacteria unkown genera OTU3	medium	0.00089022	0.089
Proteobacteria Psychrobacter	medium	0.10489744	10.4897
Fusobacteriota Cetobacterium	medium	0.00068003	0.068
Proteobacteria Pseudoalteromonas	medium	0.18818235	18.8182
Proteobacteria Psychromonas	medium	0.18077622	18.0776
Actinobacteriota Nesterenkonia	medium	0.00573697	0.5737
Proteobacteria Serratia	medium	0.19108792	19.1088
Proteobacteria Commensalibacter	medium	0.1348805	13.4881
Bacteroidota Algibacter	medium	0.01168412	1.1684
Actinobacteriota Corynebacterium	medium	0.05689981	5.69
Proteobacteria unkown genera OTU31	medium	0.04315088	4.3151
Proteobacteria Colwellia	medium	0.03083619	3.0836
Campylobacterota Pseudarcobacter	medium	0.02173617	2.1736

Proteobacteria unkown genera OTU25	medium	0.02419664	2.4197
Proteobacteria unkown genera OTU1	large	0.01326929	1.3269
Proteobacteria unkown genera OTU3	large	0.39154043	39.154
Proteobacteria Psychrobacter	large	0.0741538	7.4154
Fusobacteriota Cetobacterium	large	0.01702839	1.7028
Proteobacteria Pseudoalteromonas	large	0.00934955	0.935
Proteobacteria Psychromonas	large	0.02928561	2.9286
Actinobacteriota Nesterenkonia	large	0.3651143	36.5114
Proteobacteria Serratia	large	0.01063471	1.0635
Proteobacteria Commensalibacter	large	0.00061045	0.061
Bacteroidota Algibacter	large	0.04311716	4.3117
Actinobacteriota Corynebacterium	large	0.00557439	0.5574
Proteobacteria unkown genera OTU31	large	0.02515703	2.5157
Proteobacteria Colwellia	large	0.00571897	0.5719
Campylobacterota Pseudarcobacter	large	0.00134942	0.1349
Proteobacteria unkown genera OTU25	large	0.00809652	0.8097

**Size subset for larva only**			
Proteobacteria unkown genera OTU1	small	0.46237128	46.23713
Proteobacteria unkown genera OTU3	small	0.19624887	19.62489
Proteobacteria Psychrobacter	small	0.20744743	20.74474
Fusobacteriota Cetobacterium	small	0.03760915	3.76091
Proteobacteria Pseudoalteromonas	small	9.79E-05	0.00979
Proteobacteria Psychromonas	small	0.00086143	0.08614
Actinobacteriota Nesterenkonia	small	0	0
Proteobacteria Serratia	small	0.00015662	0.01566
Proteobacteria Commensalibacter	small	0	0
Bacteroidota Algibacter	small	0.06014331	6.01433
Actinobacteriota Corynebacterium	small	0	0
Proteobacteria unkown genera OTU31	small	0.00062649	0.06265
Proteobacteria Colwellia	small	0.00450292	0.45029
Campylobacterota Pseudarcobacter	small	0.00485532	0.48553
Proteobacteria unkown genera OTU25	small	0.02507929	2.50793
Proteobacteria unkown genera OTU1	medium	0.46352283	46.35228
Proteobacteria unkown genera OTU3	medium	0.18466249	18.46625
Proteobacteria Psychrobacter	medium	0.16359078	16.35908

Fusobacteriota Cetobacterium	medium	0.16526996	16.527
Proteobacteria Pseudoalteromonas	medium	0.00014839	0.01484
Proteobacteria Psychromonas	medium	0.00101532	0.10153
Actinobacteriota Nesterenkonia	medium	7.81E-06	0.00078
Proteobacteria Serratia	medium	2.34E-05	0.00234
Proteobacteria Commensalibacter	medium	7.81E-06	0.00078
Bacteroidota Algibacter	medium	0.01318348	1.31835
Actinobacteriota Corynebacterium	medium	0	0
Proteobacteria unkown genera OTU31	medium	0.00089035	0.08904
Proteobacteria Colwellia	medium	0.00131991	0.13199
Campylobacterota Pseudarcobacter	medium	0.0002343	0.02343
Proteobacteria unkown genera OTU25	medium	0.00612313	0.61231
Proteobacteria unkown genera OTU1	large	0.58366053	58.36605
Proteobacteria unkown genera OTU3	large	0.14549341	14.54934
Proteobacteria Psychrobacter	large	0.010543	1.0543
Fusobacteriota Cetobacterium	large	0.19800483	19.80048
Proteobacteria Pseudoalteromonas	large	4.36E-05	0.00436
Proteobacteria Psychromonas	large	0.00046535	0.04653
Actinobacteriota Nesterenkonia	large	0	0
Proteobacteria Serratia	large	1.45E-05	0.00145
Proteobacteria Commensalibacter	large	0	0
Bacteroidota Algibacter	large	0.02123142	2.12314
Actinobacteriota Corynebacterium	large	0	0
Proteobacteria unkown genera OTU31	large	7.27E-05	0.00727
Proteobacteria Colwellia	large	0.00081436	0.08144
Campylobacterota Pseudarcobacter	large	0.00061077	0.06108
Proteobacteria unkown genera OTU25	large	0.03904546	3.90455

Table S3: List of abundant bacterial genera correlated with different investigated life stages, sex, and/or body size

Actinobacteriota_unknown_genera_OTU175,adult
Actinobacteriota_unknown_genera_OTU446,adult
Actinobacteriota_unknown_genera_OTU267,adult
Actinobacteriota_unknown_genera_OTU331,adult
Actinobacteriota_unknown_genera_OTU728,adult
Actinobacteriota_unknown_genera_OTU342,adult
Actinobacteriota_unknown_genera_OTU177,adult
Actinobacteriota_unknown_genera_OTU152,adult

Actinobacteriota_Actinomycetospora,adult
Actinobacteriota_Aeromicrobium,adult
Actinobacteriota_Arthrobacter,adult
Actinobacteriota_Bifidobacterium,adult
Actinobacteriota_Blastococcus,adult
Actinobacteriota_Brachybacterium,adult
Actinobacteriota_Brevibacterium,adult
Actinobacteriota_Collinsella,adult
Actinobacteriota_Conexibacter,adult
Actinobacteriota_Corynebacterium,adult
Actinobacteriota_Cutibacterium,adult
Actinobacteriota_Dietzia,adult
Actinobacteriota_Gordonia,adult
Actinobacteriota_Hoyosella,adult
Actinobacteriota_Iamia,adult
Actinobacteriota_Kocuria,adult
Actinobacteriota_Lawsonella,adult
Actinobacteriota_Leucobacter,adult
Actinobacteriota_Libanicoccus,adult
Actinobacteriota_Mycobacterium,adult
Actinobacteriota_Nocardia,adult
Actinobacteriota_Nocardioides,adult
Actinobacteriota_Ornithinimicrobium,adult
Actinobacteriota_Pilimelia,adult
Actinobacteriota_Pseudokineococcus,adult
Actinobacteriota_Pseudonocardia,adult
Actinobacteriota_Quadrisphaera,adult
Actinobacteriota_Rathayibacter,adult
Actinobacteriota_Rhodococcus,adult
Actinobacteriota_Rothia,adult
Actinobacteriota_Rubrobacter,adult
Actinobacteriota_Streptomyces,adult
Actinobacteriota_Sva0996.marine.group,adult
Actinobacteriota_Tsukamurella,adult
Actinobacteriota_Williamsia,adult
Bacteroidota_unkonwn_genera_OTU1038,adult
Bacteroidota_unknown_genera_OTU269,adult
Bacteroidota_unknown_genera_OTU475,adult
Bacteroidota_unknown_genera_OTU432,adult
Bacteroidota_unknown_genera_OTU201,adult
Bacteroidota_unknown_genera_OTU1047,adult
Bacteroidota_unknown_genera_OTU108,adult

Bacteroidota_unknown_genera_OTU792,adult
Bacteroidota_unknown_genera_OTU118,adult
Bacteroidota_Aequorivita,adult
Bacteroidota_Algitalea,adult
Bacteroidota_Alistipes,adult
Bacteroidota_Apibacter,adult
Bacteroidota_Arenibacter,adult
Bacteroidota_Bacteroides,adult
Bacteroidota_Barnesiella,adult
Bacteroidota_Chishuiella,adult
Bacteroidota_Chryseobacterium,adult
Bacteroidota_Cryomorpha,adult
Bacteroidota_Flavobacterium,adult
Bacteroidota_Gelidibacter,adult
Bacteroidota_Gillisia,adult
Bacteroidota_Hymenobacter,adult
Bacteroidota_Kordia,adult
Bacteroidota_Marinifilum,adult
Bacteroidota_Maritimimonas,adult
Bacteroidota_Membranicola,adult
Bacteroidota_Moheibacter,adult
Bacteroidota_Myroides,adult
Bacteroidota_Nonlabens,adult
Bacteroidota_Odoribacter,adult
Bacteroidota_Parabacteroides,adult
Bacteroidota_Pedobacter,adult
Bacteroidota_Pontibacter,adult
Bacteroidota_Prevotellaceae.NK3B31.group,adult
Bacteroidota_Pricia,adult
Bacteroidota_Pseudozobellia,adult
Bacteroidota_Psychroserpens,adult
Bacteroidota_Rubrivirga,adult
Bacteroidota_Schleiferia,adult
Bacteroidota_Sphingobacterium,adult
Bacteroidota_Tenacibaculum,adult
Bacteroidota_uncultured.Bacteroidetes.bacterium,adult
Bdellovibrionota_unknown_genera_OTU985,adult
Bdellovibrionota_Peredibacter,adult
Campylobacterota_unknown_genera_OTU407,adult
Campylobacterota_Halarcobacter,adult
Campylobacterota_Sulfurimonas,adult
Campylobacterota_Sulfurospirillum,adult

Campylobacterota_Sulfurovum,adult
Chloroflexi_unknown_genera_OTU472,adult
Chloroflexi_uncultured.Chloroflexi.bacterium,adult
Cyanobacteria_unknown_genera_OTU703,adult
Cyanobacteria_unknown_genera_OTU165,adult
Cyanobacteria_unknown_genera_OTU977,adult
Cyanobacteria_unknown_genera_OTU724,adult
Cyanobacteria_unknown_genera_OTU643,adult
Cyanobacteria_unknown_genera_OTU787,adult
Cyanobacteria_Tychonema.CCAP.1459.11B,adult
Deinococcota_Truepera,adult
Desulfobacterota_unknown_genera_OTU367,adult
Desulfobacterota_Bradymonas,adult
Desulfobacterota_Desulfotalea,adult
Firmicutes_unknown_genera_OTU784,adult
Firmicutes_unknown_genera_OTU1059,adult
Firmicutes_unknown_genera_OTU807,adult
Firmicutes_unknown_genera_OTU909,adult
Firmicutes_unknown_genera_OTU669,adult
Firmicutes_unknown_genera_OTU1012,adult
Firmicutes__unknown_genera_OTU237,adult
Firmicutes_unknown_genera_OTU90,adult
Firmicutes_unknown_genera_OTU999,adult
Firmicutes_unknown_genera_OTU241,adult
Firmicutes_unknown_genera_OTU2621,adult
Firmicutes_unknown_genera_OTU263,adult
Firmicutes_.Eubacterium..eligens.group,adult
Firmicutes_.Eubacterium..hallii.group,adult
Firmicutes_.Eubacterium..ruminantium.group,adult
Firmicutes_.Ruminococcus..torques.group,adult
Firmicutes_Acetobacterium,adult
Firmicutes_Aerococcus,adult
Firmicutes_Anaerococcus,adult
Firmicutes_Anaeroplasma,adult
Firmicutes_Butyricicoccus,adult
Firmicutes_Caproiciproducens,adult
Firmicutes_Catenibacterium,adult
Firmicutes_Christensenellaceae.R.7.group,adult
Firmicutes_Clostridium.sensu.stricto.1,adult
Firmicutes_Clostridium.sensu.stricto.12,adult
Firmicutes_Coprococcus,adult
Firmicutes_Crassaminicella,adult

Firmicutes_Defluviitalea,adult
Firmicutes_Defluviitaleaceae.UCG.011,adult
Firmicutes_Dialister,adult
Firmicutes_Dolosigranulum,adult
Firmicutes_Dorea,adult
Firmicutes_Epulopiscium,adult
Firmicutes_Erysipelotrichaceae.UCG.003,adult
Firmicutes_Facklamia,adult
Firmicutes_Faecalibacterium,adult
Firmicutes_Finegoldia,adult
Firmicutes_Fusicatenibacter,adult
Firmicutes_Gemella,adult
Firmicutes_Irregularibacter,adult
Firmicutes_Lachnospira,adult
Firmicutes_Lachnospiraceae.ND3007.group,adult
Firmicutes_Lachnospiraceae.NK4A136.group,adult
Firmicutes_Lachnospiraceae.UCG.001,adult
Firmicutes_Lachnospiraceae.UCG.004,adult
Firmicutes_Lachnospiraceae.UCG.008,adult
Firmicutes_Lactiplantibacillus,adult
Firmicutes_Lactobacillus,adult
Firmicutes_Lactococcus,adult
Firmicutes_Paenibacillus,adult
Firmicutes_Paramaledivibacter,adult
Firmicutes_Peptoniphilus,adult
Firmicutes_Romboutsia,adult
Firmicutes_Roseburia,adult
Firmicutes_Ruminococcus,adult
Firmicutes_Sedimentibacter,adult
Firmicutes_Sinobaca,adult
Firmicutes_Sporosarcina,adult
Firmicutes_Streptococcus,adult
Firmicutes_Subdoligranulum,adult
Firmicutes_Turicibacter,adult
Firmicutes_UCG.002,adult
Firmicutes_UCG.003,adult
Firmicutes_UCG.005,adult
Firmicutes_UCG.008,adult
Firmicutes_uncultured.organism,adult
Firmicutes_Veillonella,adult
Fusobacteriota_Fusobacterium,adult
Gemmatimonadota_unknown_genera_OTU878,adult

Patescibacteria_unknown_genera_OTU1045,adult
Planctomycetota_unknown_genera_OTU953,adult
Proteobacteria_unknown_genera_OTU769,adult
Proteobacteria_unknown_genera_OTU487,adult
Proteobacteria_unknown_genera_OTU903,adult
Proteobacteria_unknown_genera_OTU973,adult
Proteobacteria_unknown_genera_OTU658,adult
Proteobacteria_unknown_genera_OTU597,adult
Proteobacteria_unknown_genera_OTU824,adult
Proteobacteria_unknown_genera_OTU21,adult
Proteobacteria_unknown_genera_OTU289,adult
Proteobacteria_unknown_genera_OTU978,adult
Proteobacteria_unknown_genera_OTU123,adult
Proteobacteria_unknown_genera_OTU819,adult
Proteobacteria_unknown_genera_OTU184,adult
Proteobacteria_unknown_genera_OTU20,adult
Proteobacteria_unknown_genera_OTU318,adult
Proteobacteria_unknown_genera_OTU156,adult
Proteobacteria_unknown_genera_OTU198,adult
Proteobacteria_unknown_genera_OTU731,adult
Proteobacteria_unknown_genera_OTU962,adult
Proteobacteria_unknown_genera_OTU858,adult
Proteobacteria_unknown_genera_OTU801,adult
Proteobacteria_Acidovorax,adult
Proteobacteria_Acinetobacter,adult
Proteobacteria_Actinobacillus,adult
Proteobacteria_Aeromonas,adult
Proteobacteria_Alcanivorax,adult
Proteobacteria_Alkanindiges,adult
Proteobacteria_Altererythrobacter,adult
Proteobacteria_Amaricoccus,adult
Proteobacteria_Aquabacterium,adult
Proteobacteria_Aquicella,adult
Proteobacteria_Aureimonas,adult
Proteobacteria_BD1.7.clade,adult
Proteobacteria_Bosea,adult
Proteobacteria_Bradyrhizobium,adult
Proteobacteria_Brevundimonas,adult
Proteobacteria_Burkholderia.Caballeronia.Paraburkholderia,adult
Proteobacteria_Curvibacter,adult
Proteobacteria_Devosia,adult
Proteobacteria_Enhydrobacter,adult

Proteobacteria_Erwinia,adult
Proteobacteria_Gilvimarinus,adult
Proteobacteria_Haemophilus,adult
Proteobacteria_Luteimonas,adult
Proteobacteria_Lysobacter,adult
Proteobacteria_Marimicrobium,adult
Proteobacteria_Marinicella,adult
Proteobacteria_Marinobacterium,adult
Proteobacteria_Marinospirillum,adult
Proteobacteria_Massilia,adult
Proteobacteria_Methylobacterium.Methylorubrum,adult
Proteobacteria_Neptuniibacter,adult
Proteobacteria_Novosphingobium,adult
Proteobacteria_Oceanococcus,adult
Proteobacteria_Oceanospirillum,adult
Proteobacteria_Pantoea,adult
Proteobacteria_Paraperlucidibaca,adult
Proteobacteria_Pelomonas,adult
Proteobacteria_Psychrosphaera,adult
Proteobacteria_Reinekea,adult
Proteobacteria_Reyranella,adult
Proteobacteria_Rheinheimera,adult
Proteobacteria_Roseomonas,adult
Proteobacteria_Roseovarius,adult
Proteobacteria_Salinicola,adult
Proteobacteria_Salinisphaera,adult
Proteobacteria_Sphingobium,adult
Proteobacteria_Sphingomonas,adult
Proteobacteria_Sphingopyxis,adult
Proteobacteria_Stenotrophomonas,adult
Proteobacteria_Sutterella,adult
Proteobacteria_Thermomonas,adult
Proteobacteria_uncultured.Rhizobiales.bacterium,adult
Proteobacteria_Wohlfahrtiimonas,adult
Spirochaetota_unknown_genera_OTU793,adult
Spirochaetota_Spirochaeta.2,adult
Verrucomicrobiota_Persicirhabdus,adult
X.unknown_genera_OTU633,adult
Proteobacteria_unkown_genera_OTU783,larva
Actinobacteriota_unknown_genera_OTU728,adult female
Actinobacteriota_Iamia,adult female
Bacteroidota_uncultured.Bacteroidetes.bacterium,adult female

Campylobacterota_Sulfurospirillum,adult female
Chloroflexi_uncultured.Chloroflexi.bacterium,adult female
Firmicutes_unknown_genera_OTU669,adult female
Firmicutes__unknown_genera_OTU237,adult female
Firmicutes_unknown_genera_OTU241,adult female
Firmicutes_unknown_genera_OTU1030,adult female
Firmicutes_Acetobacterium,adult female
Firmicutes_Anaeroplasma,adult female
Firmicutes_Lactiplantibacillus,adult female
Firmicutes_Lactococcus,adult female
Fusobacteriota_unknown_genera_OTU689,adult female
Proteobacteria_Erwinia,adult female
Proteobacteria_Oceanospirillum,adult female
Proteobacteria_Rheinheimera,adult female
Spirochaetota_Spirochaeta.2,adult female
Actinobacteriota_Actinomycetospora,adult male
Actinobacteriota_Libanicoccus,adult male
Actinobacteriota_Pseudokineococcus,adult male
Actinobacteriota_Sva0996.marine.group,adult male
Bacteroidota_unknown_genera_OTU1038,adult male
Bacteroidota_unknown_genera_OTU738,adult male
Bacteroidota_unknown_genera_OTU475,adult male
Bacteroidota_unknown_genera_OTU1047,adult male
Bacteroidota_unknown_genera_OTU800,adult male
Bacteroidota_Apibacter,adult male
Bacteroidota_Barnesiella,adult male
Bacteroidota_Kordia,adult male
Bacteroidota_Moheibacter,adult male
Bacteroidota_Odoribacter,adult male
Bacteroidota_Pedobacter,adult male
Bacteroidota_Prevotellaceae.NK3B31.group,adult male
Bacteroidota_Pricia,adult male
Bacteroidota_Pseudozobellia,adult male
Bacteroidota_Psychroserpens,adult male
Bdellovibrionota_.,adult male
Bdellovibrionota_Peredibacter,adult male
Campylobacterota_Sulfurovum,adult male
Cyanobacteria_unknown_genera_OTU703,adult male
Cyanobacteria_unknown_genera_OTU977,adult male
Cyanobacteria_unknown_genera_OTU787,adult male
Cyanobacteria_Tychonema.CCAP.1459.11B,adult male
Desulfobacterota_Bradymonas,adult male

Firmicutes_unknown_genera_OTU852,adult male
Firmicutes_unknown_genera_OTU909,adult male
Firmicutes_unknown_genera_OTU1012,adult male
Firmicutes_unknown_genera_OTU999,adult male
Firmicutes_unknown_genera_OTU2621,adult male
Firmicutes_Aerococcus,adult male
Firmicutes_Caproiciproducens,adult male
Firmicutes_Christensenellaceae.R.7.group,adult male
Firmicutes_Crassaminicella,adult male
Firmicutes_Finegoldia,adult male
Firmicutes_Irregularibacter,adult male
Firmicutes_Lachnospiraceae.NK4A136.group,adult male
Firmicutes_Sedimentibacter,adult male
Firmicutes_Sinobaca,adult male
Firmicutes_Turicibacter,adult male
Firmicutes_UCG.005,adult male
Firmicutes_UCG.008,adult male
Firmicutes_Veillonella,adult male
Gemmatimonadota_unknown_genera_OTU878,adult male
Proteobacteria_unknown_genera_OTU973,adult male
Proteobacteria_unknown_genera_OTU978,adult male
Proteobacteria_Acidovorax,adult male
Proteobacteria_Actinobacillus,adult male
Proteobacteria_Altererythrobacter,adult male
Proteobacteria_Aquicella,adult male
Proteobacteria_BD1.7.clade,adult male
Proteobacteria_Curvibacter,adult male
Proteobacteria_Glaciecola,adult male
Proteobacteria_Marimicrobium,adult male
Proteobacteria_uncultured.Rhizobiales.bacterium,adult male
Actinobacteriota_Libanicoccus,adult large
Bacteroidota_unknow_genera_OTU475,adult large
Bacteroidota_Apibacter,adult large
Bacteroidota_Moheibacter,adult large
Bacteroidota_Myroides,adult large
Firmicutes_unknow_genera_OTU1030,adult large
Proteobacteria_unknow_genera_OTU170,adult large
Proteobacteria_Bosea,adult large
Proteobacteria_Rheinheimera,adult large
Proteobacteria_Wohlfahrtiimonas,adult large
Spirochaetota_unknow_genera_OTU793,adult large
Bacteroidota_Kordia,adult medium

Proteobacteria_unknown_genera_OTU318,adult medium
Proteobacteria_Erwinia,adult medium
Actinobacteriota_unknown_genera_OTU331,adult small
Actinobacteriota_Actinomycetospora,adult small
Actinobacteriota_Pseudokineococcus,adult small
Bacteroidota_unknown_genera_OTU1038,adult small
Bacteroidota_unknown_genera_OTU432,adult small
Bacteroidota_unknown_genera_OTU1047,adult small
Bacteroidota_Aureispira,adult small
Bacteroidota_Hymenobacter,adult small
Bacteroidota_Odoribacter,adult small
Bacteroidota_Pedobacter,adult small
Bacteroidota_Prevotellaceae.NK3B31.group,adult small
Bacteroidota_Pricia,adult small
Bacteroidota_Psychroserpens,adult small
Bacteroidota_uncultured.Bacteroidetes.bacterium,adult small
Bdellovibrionota_Peredibacter,adult small
Cyanobacteria_unknown_genera_OTU703,adult small
Cyanobacteria_unknown_genera_OTU977,adult small
Cyanobacteria_unknown_genera_OTU787,adult small
Cyanobacteria_Tychonema.CCAP.1459.11B,adult small
Deinococcota_Truepera,adult small
Firmicutes_unknown_genera_OTU669,adult small
Firmicutes_unknown_genera_OTU1012,adult small
Firmicutes_unknown_genera_OTU999,adult small
Firmicutes_unknown_genera_OTU241,adult small
Firmicutes_Aerococcus,adult small
Firmicutes_Finegoldia,adult small
Firmicutes_Gemella,adult small
Firmicutes_Lactiplantibacillus,adult small
Firmicutes_Lactococcus,adult small
Firmicutes_Sedimentibacter,adult small
Firmicutes_Sinobaca,adult small
Firmicutes_UCG.008,adult small
Fusobacteriota_unknown_genera_OTU689,adult small
Gemmatimonadota_unknown_genera_OTU878,adult small
Planctomycetota_unknown_genera_OTU953,adult small
Proteobacteria_unknown_genera_OTU973,adult small
Proteobacteria_unknown_genera_OTU978,adult small
Proteobacteria_Actinobacillus,adult small
Proteobacteria_Altererythrobacter,adult small
Proteobacteria_Glaciecola,adult small

Proteobacteria_Marinospirillum,adult small
Proteobacteria_uncultured.Rhizobiales.bacterium,adult small
Spirochaetota_Spirochaeta.2,adult small
Actinobacteriota_Glutamicibacter,larva large
Actinobacteriota_Ilumatobacter,larva large
Bacteroidota_unknown_genera_OTU738,larva large
Bacteroidota_Aureispira,larva large
Firmicutes_unknown_genera_OTU852,larva large
Firmicutes_Anaerostipes,larva large
Firmicutes_Planococcus,larva large
Firmicutes_Staphylococcus,larva large
Proteobacteria_Phyllobacterium,larva large
Actinobacteriota_Nesterenkonia,larva medium
Bacteroidota_unknown_genera_OTU905,larva medium
Bacteroidota_Fluviicola,larva medium
Proteobacteria_unknown_genera_OTU222,larva medium
Proteobacteria_unknown_genera_OTU328,larva medium
Proteobacteria_unknown_genera_OTU103,larva medium
Proteobacteria_Cobetia,larva medium
Proteobacteria_Commensalibacter,larva medium
Proteobacteria_Oceanisphaera,larva medium
Proteobacteria_Oleispira,larva medium
Proteobacteria_Pseudomonas,larva medium
Bacteroidota_unkown_genera_OTU294,larva small
Bacteroidota_Algoriphagus,larva small
Bacteroidota_Cyclobacterium,larva small
Bacteroidota_Lutibacter,larva small
Bdellovibrionota_Halobacteriovorax,larva small
Campylobacterota_Poseidonibacter,larva small
Desulfobacterota_Desulforhopalus,larva small
Patescibacteria_unkown_genera_OTU950,larva small
Proteobacteria_unkown_genera_OTU160,larva small
Proteobacteria_Albirhodobacter,larva small
Proteobacteria_Allorhizobium.Neorhizobium.Pararhizobium.Rhizobium,larva small
Proteobacteria_Arenicella,larva small
Proteobacteria_Celeribacter,larva small
Proteobacteria_Glaciecola,larva small
Proteobacteria_Hafnia.Obesumbacterium,larva small
Proteobacteria_Hoeflea,larva small
Proteobacteria_Jannaschia,larva small
Proteobacteria_Leucothrix,larva small
Proteobacteria_Marinobacter,larva small

Proteobacteria_Paracoccus,larva small
Proteobacteria_Phaeobacter,larva small
Proteobacteria_Sphingorhabdus,larva small
Proteobacteria_Yoonia.Loktanella,larva small
Verrucomicrobiota_unkown_genera_OTU923,larva small
Verrucomicrobiota_Roseibacillus,larva small

Table S4: KO-module relative and percent abundance

KO-modules	life-stage	rel. abundance	% abundance
Archaeal proteasome	larva	0.00	0.00
MprB MprA maintenance of persistent infection two component regulatory system	larva	0.00	0.00
Xylene degradation xylene methylbenzoate	larva	0.00	0.00
Iron complex transport system	larva	0.18	17.69
ABC 2 type transport system	larva	0.10	10.48
Putative ABC transport system 2	larva	0.08	7.73
Peptides nickel transport system	larva	0.05	4.79
D Methionine transport system	larva	0.08	8.16
Lipoprotein releasing system	larva	0.08	7.68
Lipopolysaccharide export system	larva	0.06	5.94
Biotin biosynthesis pimeloyl ACP CoA biotin	larva	0.06	6.22
Biotin biosynthesis BioI pathway long chain acyl ACP pimeloyl ACP biotin	larva	0.06	6.18
acetate to acetyl CoA	larva	0.02	1.95
CMP KDO biosynthesis	larva	0.06	5.76
kdo2 lipid A synthesis	larva	0.06	5.56
pyruvate dehydrogenase complex	larva	0.03	3.32
Zinc transport system	larva	0.05	5.32
Iron III transport system	larva	0.03	3.21
**Archaeal proteasome ***	adult	0.00	0.18
**MprB MprA maintenance of persistent infection two component regulatory system ***	adult	0.00	0.32
Xylene degradation xylene methylbenzoate	adult	0.00	0.16
Iron complex transport system	adult	0.15	15.36
ABC 2 type transport system	adult	0.17	17.28
Putative ABC transport system 2	adult	0.12	11.57
Peptides nickel transport system	adult	0.08	8.29

D Methionine transport system	adult	0.05	5.30
Lipoprotein releasing system	adult	0.04	4.46
Lipopolysaccharide export system	adult	0.04	3.86
Biotin biosynthesis pimeloyl ACP CoA biotin	adult	0.03	3.48
Biotin biosynthesis BioI pathway long chain acyl ACP pimeloyl ACP biotin	adult	0.03	3.50
acetate to acetyl CoA	adult	0.06	6.31
CMP KDO biosynthesis	adult	0.03	3.45
kdo2 lipid A synthesis	adult	0.03	3.48
pyruvate dehydrogenase complex	adult	0.05	4.85
Zinc transport system	adult	0.03	3.30
Iron III transport system	adult	0.05	4.85

* unique in the subset

KO-modules	size subset larva	value	labels
tyrosine degradation phenol pathway	small	0.00	0.00
**Bacterial proteasome***	small	1.10E-05	0.001102
**Lactosylceramide biosynthesis***	small	5.51E-06	0.000551
Iron complex transport system	small	0.16	16.41
ABC 2 type transport system	small	0.13	12.69
Putative ABC transport system 2	small	0.08	8.31
Peptides nickel transport system	small	0.03	2.52
D Methionine transport system	small	0.08	7.56
Lipoprotein releasing system	small	0.08	7.85
Lipopolysaccharide export system	small	0.06	5.99
Biotin biosynthesis pimeloyl ACP CoA biotin	small	0.06	5.94
Biotin biosynthesis BioI pathway long chain acyl ACP pimeloyl ACP biotin	small	0.06	5.90
acetate to acetyl CoA	small	0.03	3.30
CMP KDO biosynthesis	small	0.06	5.80
kdo2 lipid A synthesis	small	0.06	5.83
pyruvate dehydrogenase complex	small	0.04	4.14
Zinc transport system	small	0.05	5.00
Iron III transport system	small	0.03	2.76
**tyrosine degradation phenol pathway***	medium	4.78E-06	0.000478
Bacterial proteasome	medium	0.00	0.00
Lactosylceramide biosynthesis	medium	0.00	0.00
Iron complex transport system	medium	0.18	18.13

ABC 2 type transport system	medium	0.10	9.88
Putative ABC transport system 2	medium	0.07	7.39
Peptides nickel transport system	medium	0.05	5.12
D Methionine transport system	medium	0.08	8.20
Lipoprotein releasing system	medium	0.07	7.38
Lipopolysaccharide export system	medium	0.06	5.82
Biotin biosynthesis pimeloyl ACP CoA biotin	medium	0.06	6.34
Biotin biosynthesis BioI pathway long chain acyl ACP pimeloyl ACP biotin	medium	0.06	6.24
acetate to acetyl CoA	medium	0.02	2.21
CMP KDO biosynthesis	medium	0.06	5.67
kdo2 lipid A synthesis	medium	0.05	5.44
pyruvate dehydrogenase complex	medium	0.03	3.19
Zinc transport system	medium	0.05	5.31
Iron III transport system	medium	0.04	3.66
tyrosine degradation phenol pathway	large	0.00	0.00
Bacterial proteasome	large	0.00	0.00
Lactosylceramide biosynthesis	large	0.00	0.00
Iron complex transport system	large	0.18	17.89
ABC 2 type transport system	large	0.10	9.82
Putative ABC transport system 2	large	0.08	7.87
Peptides nickel transport system	large	0.06	5.98
D Methionine transport system	large	0.09	8.57
Lipoprotein releasing system	large	0.08	8.07
Lipopolysaccharide export system	large	0.06	6.10
Biotin biosynthesis pimeloyl ACP CoA biotin	large	0.06	6.23
Biotin biosynthesis BioI pathway long chain acyl ACP pimeloyl ACP biotin	large	0.06	6.30
acetate to acetyl CoA	large	0.00	0.41
CMP KDO biosynthesis	large	0.06	5.89
kdo2 lipid A synthesis	large	0.06	5.58
pyruvate dehydrogenase complex	large	0.03	2.93
Zinc transport system	large	0.06	5.59
Iron III transport system	large	0.03	2.75

* unique in the subset

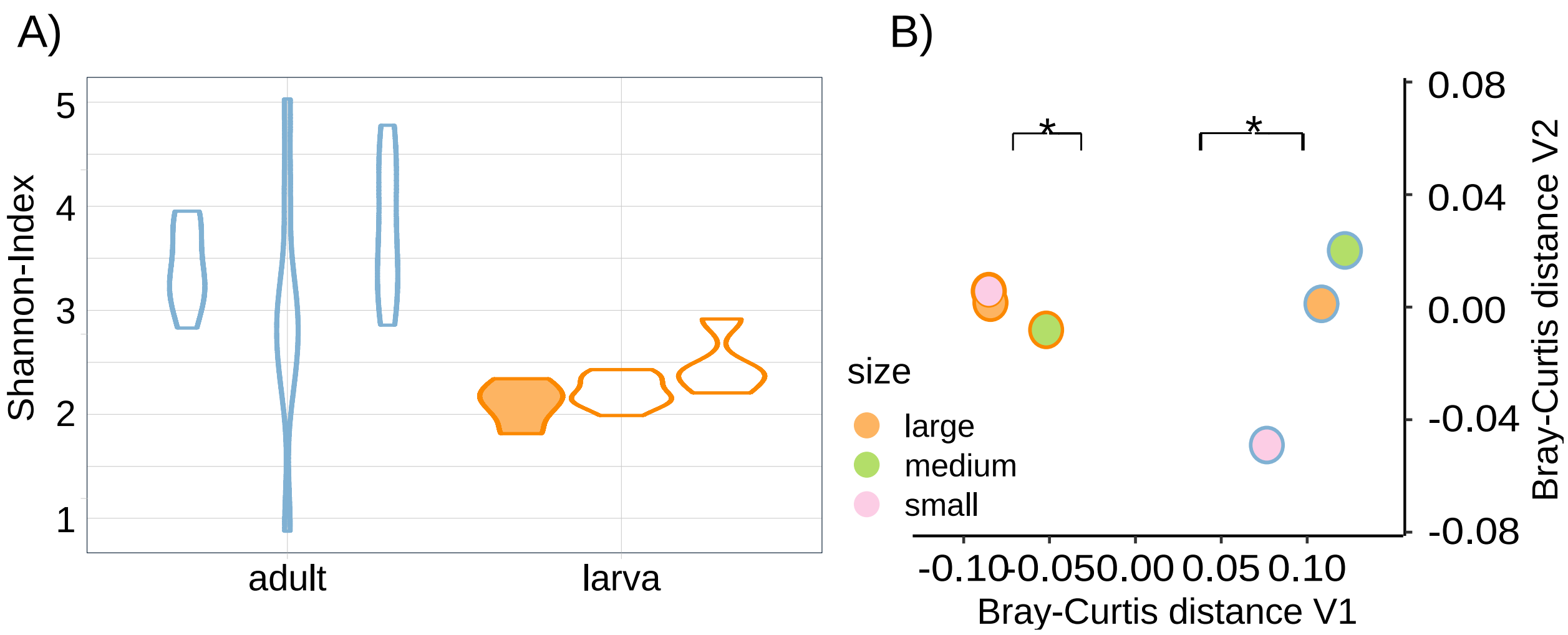

Figure S1: Comparison of diversity indices and dissimilarities between sizes in different life stages (larva and adult). (A) Shannon diversity indices show no significant difference between the sizes within the life stages, respectively (BH-FDR corrected). (B) Intersample Bray-Curtis dissimilarities reveal no significant differences between sizes for the larva and adult life stages, respectively.

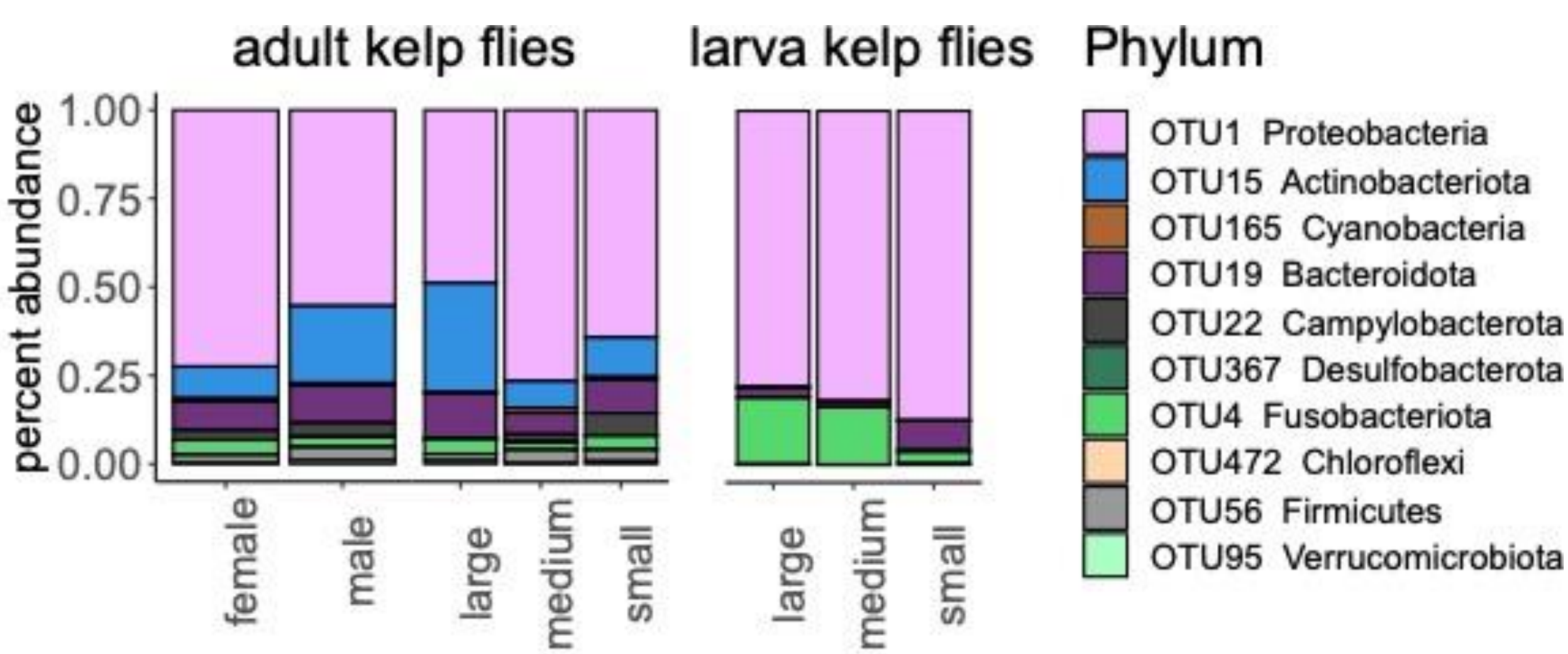

Figure S2: Phylum percent abundance abundant in adult and larval subsets categorized by sex and size. Barplots illustrate the Phylum abundance taxa for a subset of adult insects categorized

by sex (male and female) and size (small, medium, large), as well as a subset of larvae categorized by size (small, medium, large).

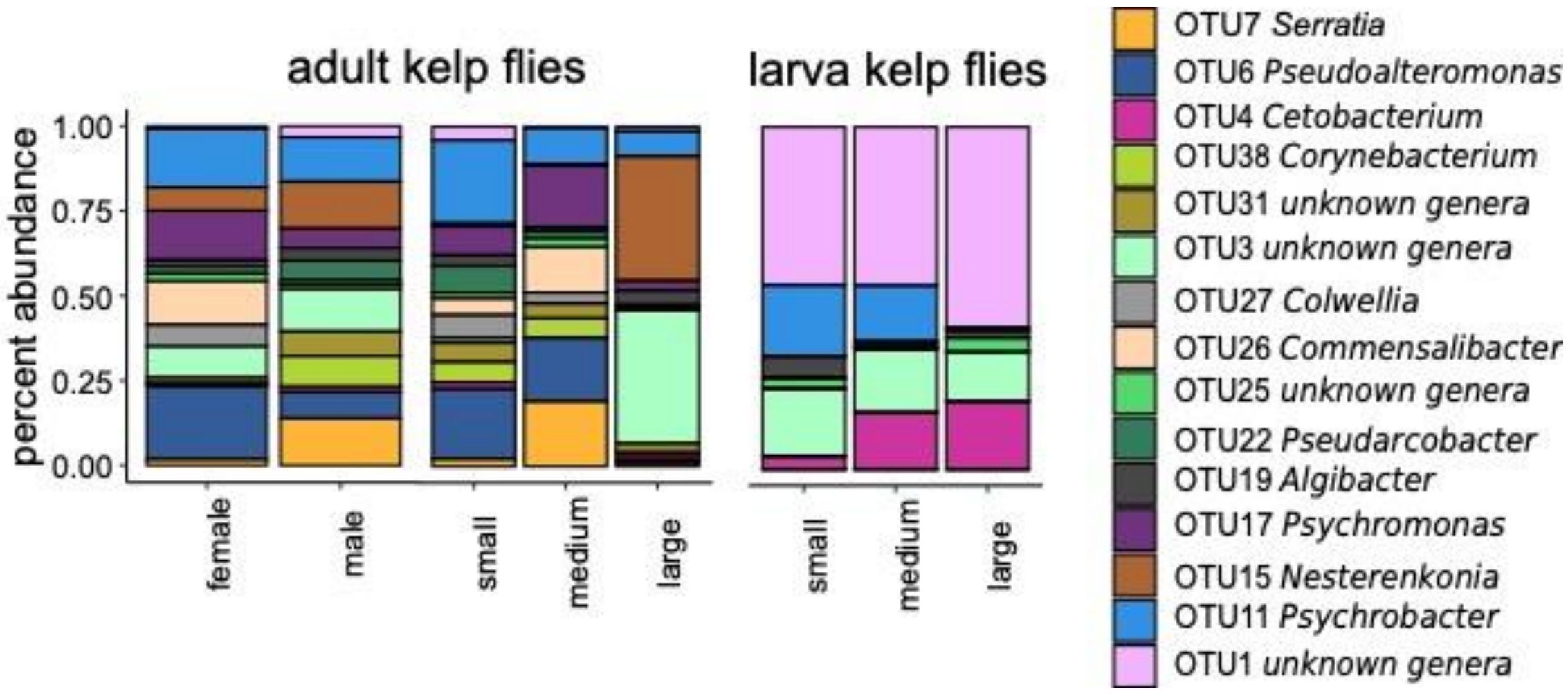

Figure S3: Top 15 most abundant taxa in adult and larval subsets categorized by sex and size. Barplots illustrate the top 15 most abundant taxa for a subset of adult insects categorized by sex (male and female) and size (small, medium, large), as well as a subset of larvae categorized by size (small, medium, large).

A) adult kelp flies

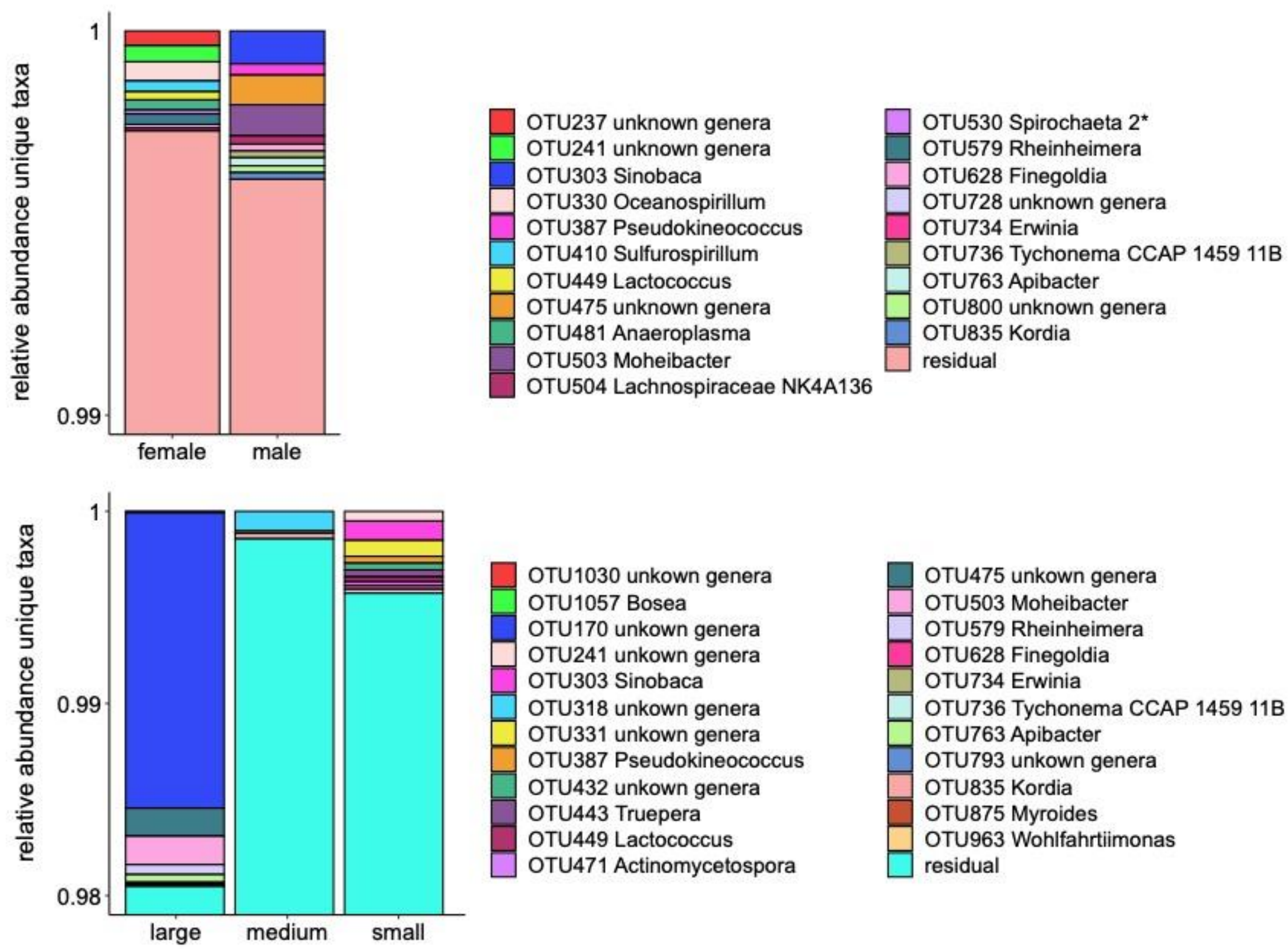

B) larva kelp flies

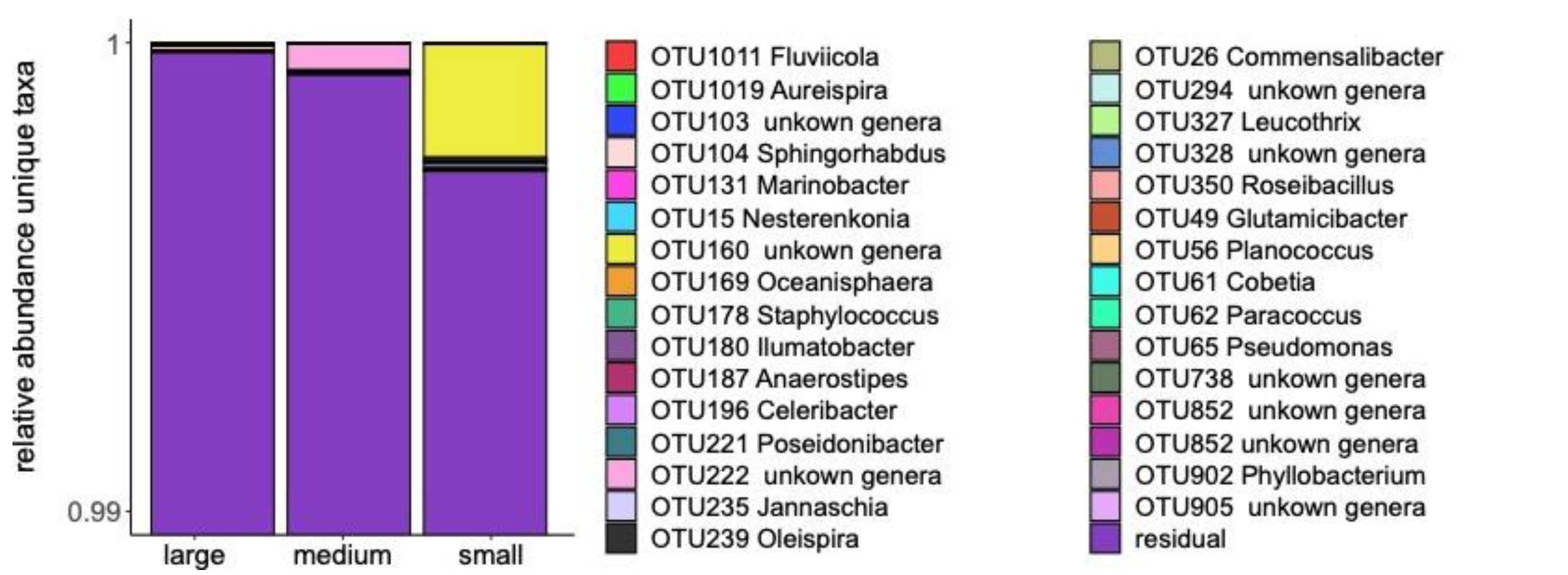

Figure S4: Abundance of top 10 unique taxa in adult and larval subsets categorized by sex and size. (A) Barplots displaying the abundance of the top 10 unique taxa for adult insects, categorized by sex (male and female) and size (small, medium, large). (B) Boxplots illustrating the abundance of the top 10 unique taxa for larval samples, categorized by size (small, medium, large).

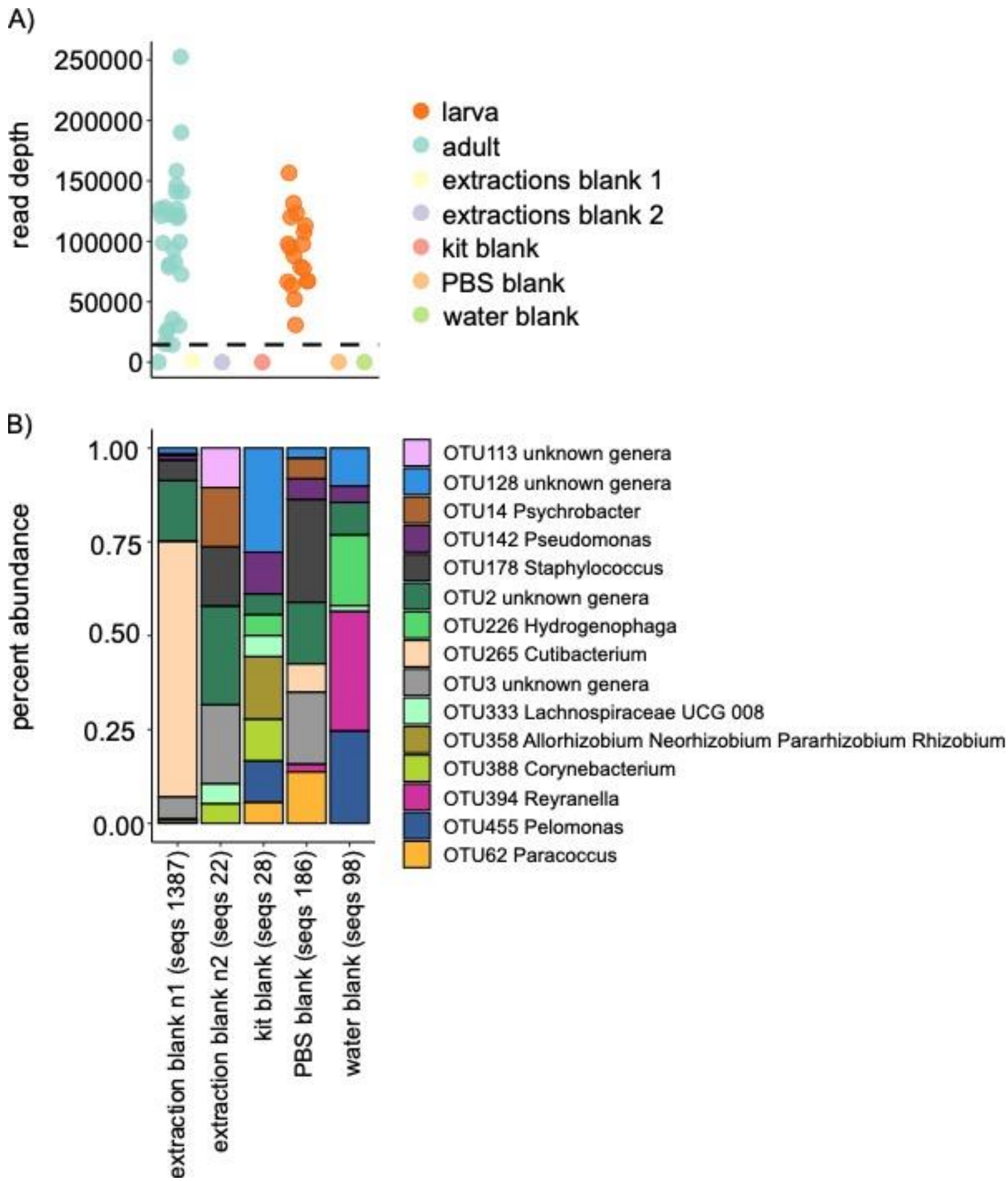

Figure S5: Read depth and abundance of the top 15 taxa in control samples. (A) Barplot illustrates the read depth of control samples (collection, extraction, and sequencing controls) compared to insect samples, with samples coloured by origin. (B) Barplot showing the top 15 most abundant taxa in the different control samples, providing insights into potential contamination or background noise in the dataset.
